# Reinvestigating the Photoprotection Properties of a Mycosporine Amino Acid Motif

**DOI:** 10.3389/fchem.2020.574038

**Published:** 2020-09-25

**Authors:** Abigail L. Whittock, Matthew A. P. Turner, Daniel J. L. Coxon, Jack M. Woolley, Michael D. Horbury, Vasilios G. Stavros

**Affiliations:** ^1^Analytical Science Centre for Doctoral Training, Senate House, University of Warwick, Coventry, United Kingdom; ^2^Department of Chemistry, University of Warwick, Coventry, United Kingdom; ^3^Molecular Analytical Science Centre for Doctoral Training, Senate House, University of Warwick, Coventry, United Kingdom; ^4^Department of Physics, University of Warwick, Coventry, United Kingdom; ^5^Diamond Science and Technology Centre for Doctoral Training, University of Warwick, Coventry, United Kingdom; ^6^School of Electronic and Electrical Engineering, University of Leeds, Leeds, United Kingdom

**Keywords:** photoprotection, photostability, ultrafast, spectroscopy, mycosporine

## Abstract

With the growing concern regarding commercially available ultraviolet (UV) filters damaging the environment, there is an urgent need to discover new UV filters. A family of molecules called mycosporines and mycosporine-like amino acids (referred to as MAAs collectively) are synthesized by cyanobacteria, fungi and algae and act as the natural UV filters for these organisms. Mycosporines are formed of a cyclohexenone core structure while mycosporine-like amino acids are formed of a cyclohexenimine core structure. To better understand the photoprotection properties of MAAs, we implement a bottom-up approach by first studying a simple analog of an MAA, 3-aminocyclohex-2-en-1-one (**ACyO**). Previous experimental studies on **ACyO** using transient electronic absorption spectroscopy (TEAS) suggest that upon photoexcitation, **ACyO** becomes trapped in the minimum of an S_1_ state, which persists for extended time delays (>2.5 ns). However, these studies were unable to establish the extent of electronic ground state recovery of **ACyO** within 2.5 ns due to experimental constraints. In the present studies, we have implemented transient vibrational absorption spectroscopy (as well as complementary TEAS) with Fourier transform infrared spectroscopy and density functional theory to establish the extent of electronic ground state recovery of **ACyO** within this time window. We show that by 1.8 ns, there is >75% electronic ground state recovery of **ACyO**, with the remaining percentage likely persisting in the electronic excited state. Long-term irradiation studies on **ACyO** have shown that a small percentage degrades after 2 h of irradiation, plausibly due to some of the aforementioned trapped **ACyO** going on to form a photoproduct. Collectively, these studies imply that a base building block of MAAs already displays characteristics of an effective UV filter.

## Introduction

Ultraviolet (UV) radiation, in particular UVA (315–400 nm) and UVB (280–315 nm), that reaches the Earth's surface has both positive and negative effects on living organisms (Holick, 2004; Lucas et al., 2006; Humble, 2010). Consequently, nature has developed its own photoprotection to safeguard from harmful DNA damage. In humans, this natural photoprotection is achieved by producing melanin pigments that can absorb UVA and UVB radiation before it reaches DNA (Kollias et al., 1991). However, as melanin production is a delayed process and does not absorb all UVA and UVB radiation that reaches the skin, a more immediate form of protection is required, i.e., sunscreens (Eller and Gilchrest, 2000; Brenner and Hearing, 2008; Wang and Lim, 2016). Some UV filters found in sunscreens have been linked to the cause of damaging environmental effects as well as adverse dermatological effects (Bryden et al., 2006; Danovaro et al., 2008; Downs et al., 2013, 2016; Warshaw et al., 2013; Schaap and Slijkerman, 2018). As a result, there is an increased need for the identification of new UV filters that are less harmful to both the environment and humans. Drawing inspiration from nature offers a promising solution to these negative impacts. One approach is to base candidate UV filters off structures observed in plants and microorganisms (Bandaranayake, 1998; Dean et al., 2014; Baker et al., 2016, 2018; Horbury et al., 2017, 2018, 2019; Luo et al., 2017; Zhao et al., 2019a,b).

Cyanobacteria, fungi, macro- and microalgae all synthesize a family of molecules termed mycosporines and mycosporine-like amino acids (Sinha et al., 2007; Balskus and Walsh, 2010). In the literature, mycosporines and mycosporine-like amino acids are used synonymously. However, mycosporines are a family of molecules comprised of a cyclohexenone core and mycosporine-like amino acids are comprised of a cyclohexenimine core (herein both mycosporines and mycosporine-like amino acids will be termed MAAs) (Gao and Garcia-Pichel, 2011). MAAs present a strong absorbance band in the UVA and UVB, and their high photostability makes them highly desirable as potential UV filters (Bandaranayake, 1998; Conde et al., 2000, 2007; Sinha et al., 2000; Moliné et al., 2011; Rastogi and Incharoensakdi, 2014). As the extraction from natural sources and the synthetic preparation of natural MAAs produces small quantities, some work has been carried out on synthetic MAA motifs as an alternative; these molecules have also demonstrated promising levels of photostability thus far (White et al., 1989, 1995; Bandaranayake, 1998; Losantos et al., 2017, 2019; Woolley et al., 2018). Complementary studies, implementing computational and ultrafast spectroscopic techniques have been conducted on various MAA motifs in an effort to guide future UV filter design through knowledge of the photoprotection mechanisms (Losantos et al., 2017, 2019; Woolley et al., 2018). Their finding will be briefly outlined here.

Losantos et al. (2017) implemented a CASPT2/CASSCF methodology to evaluate the minimum energy paths of a selection of MAA motifs composed of either a cyclohexenone or a cyclohexenimine core. Their results showed that a cyclohexenone core is potentially a poor scaffold for sunscreen applications, as following photoexcitation to the lowest optically bright S_2_ excited state, a geometric distortion to a non-planar geometry leads to relaxation to the S_1_ state via a S_2_/S_1_ conical intersection (CI). The electronic excited state population then traverses along the potential energy surface of the S_1_, before becoming trapped in a minimum on the S_1_ potential energy surface. For the cyclohexenimine core, Losantos et al. (2017) proposed that after initial photoexcitation to the lowest optically bright S_1_ excited state, fast relaxation along an out-of-plane geometry distortion leads to an accessible S_1_/S_0_ CI, resulting in efficient repopulation of the electronic ground state. These results by Losantos et al. (2017) are corroborated by computational studies on the natural MAAs palythine and porphyra-334 (Sampedro, 2011; Koizumi et al., 2017; Hatakeyama et al., 2019). Interestingly, a computational study on gadusol (a molecule closely related to MAAs with a cyclohexenone core) found an accessible barrierless S_1_/S_0_ CI (Losantos et al., 2015). This, in addition to experimental studies on gadusol which reported high levels of photostability and rapid non-radiative decay as the dominant relaxation pathway (Arbeloa et al., 2011), indicates that molecules with a cyclohexenone core do display a number of ideal properties of a UV filter. Therefore, there is still much to learn about these cyclohexenone systems.

**Figure 1:**
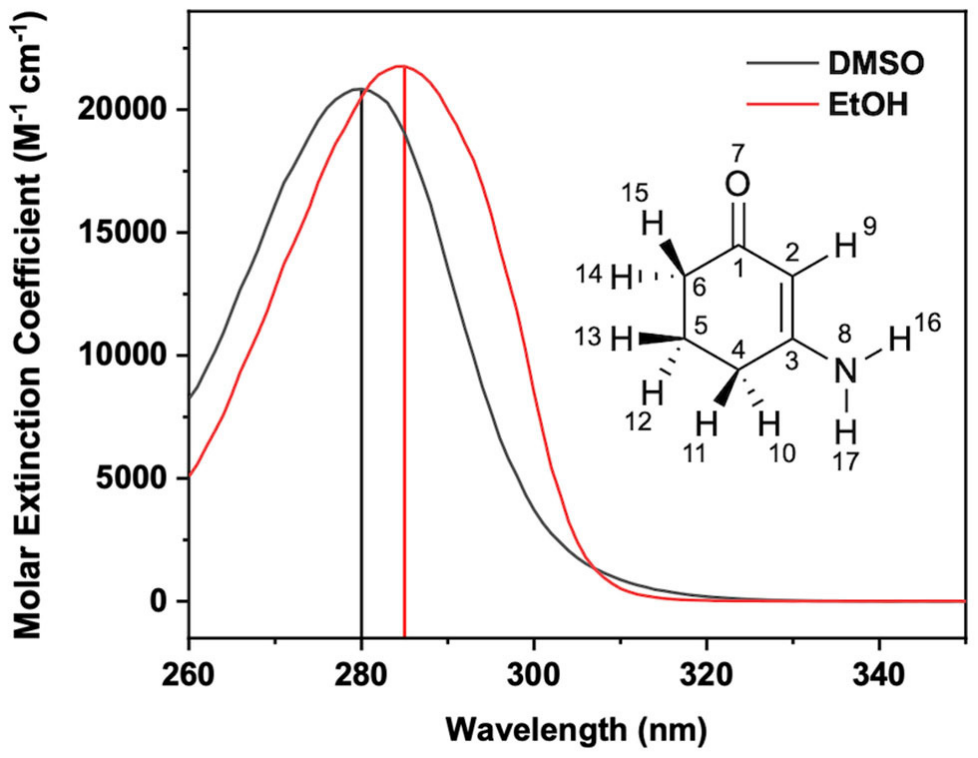
Steady-state UV/visible extinction spectra of **ACyO** in EtOH (black line) and DMSO (red line). Vertical line corresponds to the absorption maxima of each solution; 285 nm for EtOH and 280 nm for DMSO. Inset is the structure of **ACyO** along with its atom numbers.

Woolley et al. (2018) and Losantos et al. (2019) have conducted transient electronic absorption spectroscopy (TEAS) experiments on MAA motifs to unravel their photoprotection mechanisms. Woolley et al. (2018) investigated two MAA motifs, the one of interest for this paper being 3-aminocyclohex-2-en-1-one (**ACyO**), see inset of Figure 1 for the structure of **ACyO**. Their TEAS experimental results for **ACyO** in methanol (polar protic) and acetonitrile (polar aprotic) demonstrated a persistent excited state absorption (ESA), >2.5 ns, corroborating calculations from Losantos et al. (2017) that (at least a fraction of) **ACyO** becomes trapped in a minimum of the S_1_. We note here that the calculated barrier to the S_1_/S_0_ CI is 0.04 eV for **ACyO** in the gas-phase (Woolley et al., 2018). Whilst population persisted in the excited state for nanoseconds, no real indication as to the percentage of **ACyO** that remained in the electronic excited state can be inferred from the TEAS experiment. This is due to TEAS experimental limitations as Woolley et al. (2018) were unable to observe a ground state bleach (GSB) of **ACyO**. Consequently, they could not establish the extent of electronic ground state recovery which would give a direct measure of the photostability of **ACyO**. Herein, we propose that UV pump infrared probe transient vibrational absorption spectroscopy (TVAS) can be used to measure the extent of vibrational ground state recovery in the electronic ground state on ultrafast timescales after photoexcitation, thus providing insight into the overall electronic ground state recovery of **ACyO**.

To better understand the behavior of MAA motifs with a cyclohexenone core, we have performed TVAS, along with complementary TEAS and long-term irradiation studies on **ACyO**, to study the GSB recovery of its vibrational modes. We build on a study by Sui et al. (2012) who used density functional theory (DFT) to assign the vibrational modes of **ACyO** in acetonitrile and water; for reasons that will become apparent later, our experiments utilized ethanol (EtOH) and dimethyl sulfoxide (DMSO) as solvents. In order to assign the vibrational modes both in the electronic ground and excited state in EtOH and DMSO, we have implemented implicit- and explicit-solvent DFT and time-dependent DFT (TDDFT) (Casida, 1995) calculations using a similar level of theory to Sui et al. (2012). These high-level calculations were necessary for the identification of any overlapping electronic ground and excited state frequencies that would influence the interpretation of our TVAS data. We report in the present work that TVAS is a powerful technique that can extract GSB recovery quantum yields for the vibrational modes of a molecule on ultrafast timescales by comparing the extent of the bleach recovery with respect to the initial GSB. Previous publications have reported GSB recovery quantum yields, and how such qualitative information provides valuable insight into the efficiency of relaxation pathways (Holm et al., 2003; Rini et al., 2003; Murdock et al., 2014, 2015). For **ACyO**, the resultant quantum yields in addition to its overall photostability demonstrates promising properties that are required of a UV filter, providing some evidence for the photoprotective capabilities of MAAs with a cyclohexenone core.

## Materials and Methods

**ACyO** was purchased from Alfa Aesar (95% purity) and solutions of ~40 μM, 4 mM, and 50 mM concentrations were made up in the two solvents used in this study. EtOH (≥99.8%) and DMSO (≥99.9%) were purchased from VWR Chemicals and Fisher Scientific, respectively.

### Steady-State Spectroscopy

**ACyO** samples in EtOH and DMSO of concentrations ~40 μM contained in a 1 cm path length quartz cuvette were irradiated within a solar simulator (91,191–1,000, Oriel Instruments) which has an output power equivalent to the sun at the Earth's surface (~1,000 W/m^2^) for 7,200 s (120 min, 2 h). At various time intervals, UV/visible spectra were recorded using a UV/visible spectrometer (Cary 60, Agilent Technologies). To confirm that the degradation occurring was due to photodegradation, UV/visible spectra of **ACyO** solutions that had not been irradiated were taken before and after 7,200 s.

Fourier transform infrared (FTIR) spectra were obtained using a FTIR spectrometer (VERTEX 70v, Bruker) under a nitrogen environment to remove vibrational modes associated with atmospheric gases. 50 mM **ACyO** solutions in EtOH and DMSO were contained within a demountable liquid cell (Harrick Scientific Products Inc.) with a path length of 100 μm achieved by inserting 100 μm PTFE spacers between two CaF_2_ windows (front window 1 mm and back window 2 mm thickness). The FTIR spectra were recorded over an energy range of 500–4,000 cm^−1^ with a resolution of 1 cm^−1^.

### Ultrafast Spectroscopy

TEAS and TVAS measurements were taken at the Warwick Center for Ultrafast Spectroscopy (WCUS: www.go.warwick.ac.uk/fac/sci/wcus). The setup of the TEAS experiment has been previously reported (Woolley et al., 2018), but it will be briefly described here. 4 mM solutions of **ACyO** in EtOH and DMSO were circulated through a demountable liquid cell with a path length of 100 μm set up in the same way as for FTIR spectroscopy described above. The samples were continuously circulated through the cell using a diaphragm pump (SIMDOS 02) to ensure a fresh sample was interacting with each laser shot. 800 nm pulses (12 W, 1 kHz, 40 fs) were generated by a Ti:Sapphire regenerative amplified laser system (Dual Ascend Pumped Spitfire Ace, Spectra-Physics) seeded by a Mai Tai (Spectra-Physics). The beam was split into four fractions, each fraction having its own recompression grating. One of the four fractions (3.5 W) was split into two further fractions in order to generate the pump and probe beams required for the TEAS experiments.

The first of the fractions (2.5 W) seeds an optical parametric amplifier (Topas-Prime with UV-extension, Light Conversion) which allows for a tuneable pump beam wavelength that includes 285 and 280 nm for photoexciting **ACyO** in EtOH and DMSO, respectively. These wavelengths were chosen as they correspond to the absorption maximum of each **ACyO** solution as displayed in Figure 1. The pump beam was set to a power of ~500 μW and was focused beyond the sample holder to give a beam diameter of 500 μm at the sample holder. The probe beam was generated by focussing 5% of the remaining 1 W fundamental 800 nm beam which had been further attenuated and irised on to a vertically translated CaF_2_ window (2 mm thickness) to generate a white light continuum (320–720 nm). The probe pulse polarization was held at the magic angle (54.7°) with regards to the pump pulse polarization; this is done to avoid dynamical contributions from molecular reorientations. The path length of the 800 nm beam used to generate the probe beam can be varied to give pump-probe time delays (Δ*t*) of −1 ps to 2.5 ns using a gold retroreflector mounted on a motorized optical delay line. Before reaching the sample, the pump beam passes through an optical chopper operating at a repetition rate of 500 Hz, blocking every other pulse of the 1 kHz pulse train. This allows for direct comparison of the signal detected by a fiber-coupled spectrometer (AvaSpec-ULS1650F, Avantes) for the pumped and unpumped samples which is displayed as changes in optical density (ΔOD) in the resultant transient electronic absorption (TEA) spectra.

Pump power dependency studies were carried out to ensure that ΔOD was linearly dependent on photon flux (see Supplementary Figure 1). Chirp correction of the TEA spectra was achieved using the KOALA package (Grubb et al., 2014). Global fitting of the TEA spectra was employed using the software package Glotaran (Mullen and Van Stokkum, 2007; Snellenburg et al., 2012), and a sequential kinetic model ($\text{A}\overset{\tau_{e1}}{\to }\text{B}\overset{\tau_{e2}}{\to }\text{C}\ldots$) was used over the entire spectral region of our probe (320–720 nm). The evolution associated difference spectra for the time constants of each fit can be found in Supplementary Figure 2. The quality of the fits was assessed through the associated residuals, see Supplementary Figure 2. The instrument response function, accounting for the temporal resolution of our TEAS measurements, was determined by fitting a Gaussian over the time zero artifacts of solvent-only scans and taking the full width half-maximum, see Supplementary Figure 3 for the instrument response functions of EtOH and DMSO.

For the TVAS set up, 50 mM **ACyO** solutions in EtOH and DMSO were circulated through a demountable liquid cell the same way as reported above for the TEAS set-up. The pump beam was set to a higher power of 700 μW for the TVAS experiments. The probe beam (240 μW, 360 μm beam diameter) for the TVAS experiment was generated by seeding a second optical parametric amplifier (Topas-C, Spectra-Physics) with a second 3.5 W fraction of the fundamental 800 nm beam. The generated probe beam allows for a tuneable IR wavelength that includes 6,420 and 6,289 nm which were the wavelengths used in this experiment for **ACyO** in EtOH and DMSO, respectively. The path length of the probe beam was varied in a similar way to the TEAS set-up to give Δ*t* of −1 ps to 1.8 ns. Atmospheric absorption lines were excluded by purging the probe line with nitrogen gas. Before the probe beam arrived at the sample compartment, it was incident on a CaF_2_ beamsplitter where it was split equally in to reference and probe pulses. The reference pulse did not traverse the sample and was detected for the subtraction of shot-to-shot laser noise. The probe pulse passed through the sample where it was partially absorbed before entering an imaging spectrometer (iHR320, HORIBA Scientific). Once in the spectrometer, the probe was dispersed by a diffraction grating (6 μm blaze, 100 lines mm^−1^ resolution) onto a mercury cadmium telluride (MCT) detector array (FPAS-0144, Infrared Systems Development) which was made up of two 64-pixel linear arrays—one each for the probe and reference pulses. The MCT detector was cooled using liquid nitrogen to eliminate thermal contributions to the signal. Like in the TEAS experiment, the pump beam passes through an optical chopper operating at a repetition rate of 500 Hz, blocking every other pulse of the 1 kHz pulse train. This allows for direct comparison of the signal for the pumped and unpumped samples which is displayed as ΔOD in the resultant transient vibrational absorption (TVA) spectra.

We converted between pixel number and wavelength using a mid-IR polystyrene calibration card (Perkin Elmer) as our reference. Similarly to the TEAS, pump power dependency studies were carried out, see Supplementary Figure 4. Only ~100 cm^−1^ windows were investigated for each solvent environment; this was dictated by the spectral congestion emanating from the solvent and the transition intensity of **ACyO** vibrational modes. Exponential fits of the GSB features were employed to extract time constants associated with the dynamical processes **ACyO** undergoes upon photoexcitation.

### Computational Methods

Vibrational frequencies in the electronic ground (S_0_) and excited (S_1_) state were predicted for **ACyO** in both implicit- and explicit-solvent environments for EtOH and DMSO. The NWChem package (Valiev et al., 2010) was used to perform DFT and TDDFT calculations. These were done at the PBE0/cc-pVTZ level of theory (Davidson, 1996; Perdew et al., 1996; Adamo and Barone, 1999). Implicit-solvent calculations were employed using the COSMO solvent model for both EtOH and DMSO which models each solvent's dielectric parameters (Klamt and Schüürmann, 1993; Winget et al., 1999; York and Karplus, 1999). We add that the level of theory described has previously been used for a similar system studied by Turner et al. (2019) which also performed vibrational frequency calculations. Due to discrepancies in the calculated S_0_ vibrational frequencies and their corresponding vibrational modes, in addition to the fact EtOH and DMSO interact strongly with **ACyO**, it was determined that explicit-solvent contributions should be considered. See Supplementary Table 1 and Supplementary Figure 5 for implicit-solvent vibrational frequency results. This high-level of theory has been used so that accurate electronic excited state frequencies are predicted, which in turn provides valuable insight into any overlapping electronic ground and excited state frequencies in the TVAS probe region. As a result, reliable GSB recovery quantum yields can be extracted from the TVA spectra. Although neither implicit- or explicit-solvent results are perfect, the scaling factors required for explicit-solvent (0.997 and 0.982 for EtOH and DMSO, respectively) were significantly closer to 1 than implicit-solvent (0.976 for both solvents) indicating improved accuracy for the explicit-solvent calculations. Therefore, the decision was made to report explicit-solvent herein. Further to this, the asymmetry of the 1.8 ns lineout from our TVA spectra can be explained by the computed S_1_ frequencies determined by EtOH explicit-solvent (see later discussion). On the other hand, the computed S_1_ frequencies for EtOH implicit-solvent do not provide such clarity. Furthermore, regardless of whether an implicit- or explicit-solvent model is used for **ACyO** in DMSO, there are no overlapping S_1_ frequencies with the probed peaks in the TVAS experiment.

Solvent shells were generated through a similar method used by Zuehlsdorff et al. (2017) and Turner et al. (2019). In this, a classical molecular dynamics simulation was performed using the AMBER package (Case et al., 2015). **ACyO**, optimized in DFT at the level of theory discussed previously, was immersed in a 20 Å cube of explicit-solvent (both EtOH and DMSO). The system was heated over 20 ps in the NVT ensemble wherein the temperature is raised from 0 to 300 K. This, along with all further calculations, was achieved using a Langevin thermostat with a collision frequency of 1 ps^−1^. Following this, a 400 ps pressure equilibration is utilized in the NPT isothermic-isobaric ensemble with the pressure fixed at 1 atm. Next, the system was equilibrated at a constant temperature of 300 K for 100 ps. Finally, snapshots were generated via a further NVT ensemble run, this time with a fixed temperature of 300 K for 8 ns. 2000 snapshots were extracted by recording a frame every 4 ps. By saving every tenth snapshot, 200 of these snapshots were exported. Within each snapshot, the solute and hydrogen bonding solvent molecules were extracted by removing any solvent molecules that were further than 1.5 Å from the solute.

As has been previously conducted by Turner et al. (2019), four snapshots were chosen for each solvent. Each snapshot has a different solvent environment and initial structures are shown in Supplementary Table 2. In the case of DMSO, only two solvent molecules were within 1.5 Å of **ACyO** and hence the four chosen snapshots were selected to ensure that the DMSO molecules were in different orientations in each snapshot (see Supplementary Table 2). For EtOH, a variety of solvent environments over the 200 snapshots were found, the most common being; one EtOH on the carbonyl oxygen and one EtOH on the amine hydrogen, two EtOH on the carbonyl oxygen and one EtOH on the amine hydrogen, one EtOH on the carbonyl oxygen and two EtOH on the amine hydrogens and finally two EtOH on the carbonyl oxygen and two EtOH on the amine hydrogens. One of each environment was selected for the calculations in order to evaluate the different solvent environments determined by molecular dynamics (see Supplementary Table 2). Calculations of full solvent shells were not conducted for the present work. This is due to previous findings by Turner et al. (2019) who found that full solvent shell calculations and smaller clusters with only two solvent molecules did not generate notable differences in the calculated vibrational frequencies. Furthermore, such calculations take significant computational expenditure due to the larger number of atoms.

The DFT calculations that were conducted were as follows: initially a geometry optimisation was employed on all the implicit- and explicit-solvent structures stated above followed by an electronic ground state frequency calculation. Vertical excitations were conducted and the results corroborated previous work stating that the first optically bright state is the S_2_ (Sui et al., 2012; Losantos et al., 2017). Following this calculation, electronic excited state geometry relaxation calculations were carried out to determine the relaxed geometry on the S_1_ excited state. The S_1_ was chosen based on previous work that computed the minimum energy path and found that **ACyO** becomes trapped in the minimum of its S_1_ excited state (Losantos et al., 2017; Woolley et al., 2018). Finally, electronic excited state vibrational frequency calculations were conducted on the S_1_ relaxed geometry. Only averaged explicit-solvent vibrational frequencies for **ACyO** in EtOH and DMSO are presented herein, but all individual results for the implicit- and explicit-solvent environments can be found in the Supplementary Material including the optimised geometries and vertical excitation results (see Supplementary Tables 1–5). We also note that all reported frequencies are between 1,500 and 1,750 cm^−1^ as it is the region of interest for the present work and extensive evaluation of the computed frequencies is outside the scope of this work.

Scaling factors were applied to the calculated S_0_ and S_1_ frequencies and were calculated by using one experimental peak as a reference so that the calculated frequency exactly matches the reference experimental peak. This method is common practice and has been employed in a similar study by Turner et al. (2019) and Grieco et al. (2018). The chosen S_0_ reference peaks were 1,559 and 1,577 cm^−1^ for **ACyO** in EtOH and DMSO, respectively, and were chosen because they were the strongest experimental peaks observed between 1,500 and 1,750 cm^−1^. As there were no clear experimental S_1_ frequencies obtained in this study, we tentatively applied the same scaling factor to the S_1_ frequency calculation as was applied to the S_0_ frequency calculation for both solvent environments; a similar approach was implemented by Baker et al. (2015). The resultant scaling factors for the averaged explicit-solvents snapshots of **ACyO** in EtOH and DMSO were 0.997 and 0.982, respectively.

## Results and Discussion

**Figure 2:**
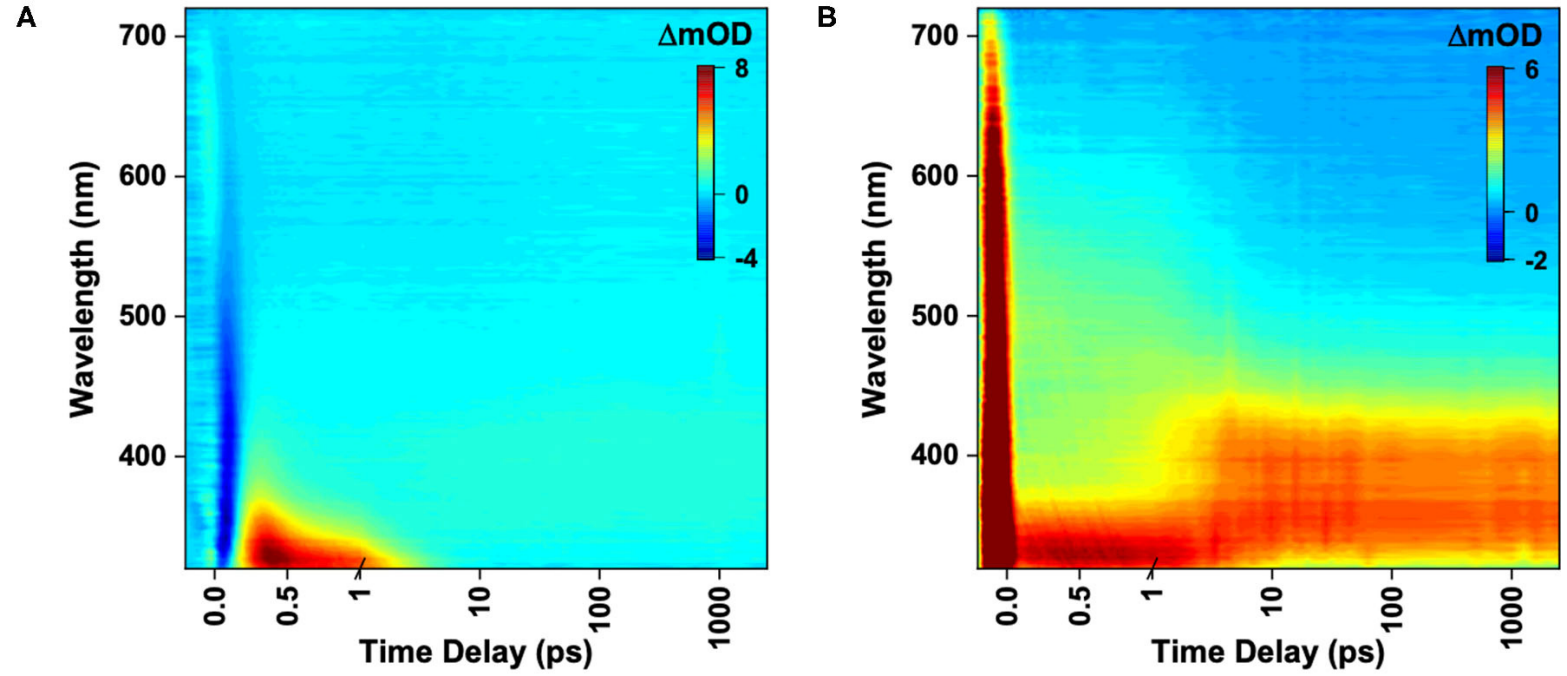
TEA spectra represented as false color maps of 4 mM **ACyO** in **(A)** EtOH photoexcited at 285 nm and **(B)** DMSO photoexcited at 280 nm. For **(A,B)** the time delays are plotted linearly until 1 ps and then as a log scale from 1 to 2,500 ps.

We first take a look at the TEA spectra acquired for **ACyO** in EtOH and DMSO which are displayed as false color maps in Figure 2; for lineouts of the TEA spectra see Supplementary Figure 6. The choice to use EtOH and DMSO as solvents was firstly to demonstrate dynamics in different solvent types (protic and aprotic), and secondly because these solvents displayed clear spectral windows in the region of interest for our FTIR and TVAS experiments. We note here that our TEA spectra strongly correlate with the findings of Woolley et al. (2018); the **ACyO** excited-state dynamics in EtOH are very similar to those observed in methanol (as might be expected; both polar and protic), and the **ACyO** excited state dynamics in DMSO are also very similar to those observed in acetonitrile (both polar and aprotic). Hence only a brief discussion is required here. Starting with **ACyO** in EtOH shown in Figure 2A, we observe a negative feature at early pump-probe time delays (Δ*t* < 0.2 ps) identified as multiphoton initiated dynamics (see Supplementary Figure 1), followed by an intense ESA centered at ~330 nm which blue-shifts out of the probe window and mostly decays by ~5 ps. A second ESA of less intensity grows in within 5 ps centered at ~400 nm and persists to the final time delay of our experiment (Δ*t* = 2.5 ns), see Supplementary Figure 6A for lineouts. We now take a look at the TEA spectra for **ACyO** in DMSO shown in Figure 2B. At early time delays, there is a broad ESA spanning from 320 to 720 nm peaking at ~330 nm. The broad ESA narrows and the greatest intensity red-shifts to center at ~370 nm in around 5 ps. This feature then persists to the maximum Δ*t* of the experiment (Δ*t* = 2.5 ns), see Supplementary Figure 6B for lineouts.

Although mentioned previously, it is worth revisiting the proposed cyclohexenone core deactivation pathway upon photoexcitation reported by Losantos et al. (2017) as it is the basis for the time constant assignment presented herein. The vertical excitation calculations that were carried out for the present study corroborate previous work which states that **ACyO** is initially photoexcited to the S_2_ through a $\pi_{\text{L}}^{*}\leftarrow \pi_{\text{H}}$ transition (see Supplementary Table 4 and Supplementary Figure 7) (Sui et al., 2012; Losantos et al., 2017). The computed minimum energy path demonstrates that a similar molecule to **ACyO** (NHCH_3_ substituent on C_3_, herein termed the NHCH_3_ molecule, see inset of Figure 1) studied by Losantos et al. (2017) redistributes its energy after photoexcitation causing a geometric distortion. This leads to an accessible S_2_/S_1_ CI where it then vibrationally cools to a minimum on the S_1_. Woolley et al. (2018) conducted complementary calculations on **ACyO** finding that the calculated energy barrier to the S_1_/S_0_ CI for **ACyO** in the gas phase is 0.04 eV (Woolley et al., 2018), *cf*. 0.2 eV for the NHCH_3_ molecule studied by Losantos et al. (2017). We now assign the dynamical processes to the extracted time constants from the global sequential fit, re-evaluating our previous work in light of the present and previous data.

Global fitting of **ACyO** in EtOH extracted three time constants and can be found in Table 1; we add that as the negative feature is the result of multiphoton initiated dynamics, it has been omitted from the global fit. We propose that the first time constant, τ_e1_ = 0.24 ps, corresponds to the initial geometry relaxation and solvent rearrangement **ACyO** undergoes in order to traverse the S_2_/S_1_ CI and populate the S_1_ potential energy surface. We attribute the second time constant, τ_e2_ = 1.2 ps, with the vibrational cooling of **ACyO**, via vibrational energy transfer (both intramolecular and intermolecular), as it samples the S_1_ potential energy surface before overcoming the barrier to, and traversing through the S_1_/S_0_ CI. From the evolution associated difference spectra and lineouts of **ACyO** in EtOH (see Supplementary Figures 2A, 6A), narrowing of the ESA is observed which is a good indication that vibrational cooling on the S_1_ potential energy surface is occurring (also the case for **ACyO** in DMSO, see Supplementary Figures 2B, 6B). We note here that as the ESA (~330 nm) decays out of the probe window in our TEA spectra, it is possible that τ_e2_ is an underestimation of the time constant associated with repopulation of the electronic ground state. The final time constant, τ_e3_ > 2.5 ns, corresponds to any **ACyO** that has remained trapped in the minimum of the S_1_ excited state.

**Table 1:** Time constants and associated errors extracted from the sequential global fit of the TEA spectra for **ACyO** in EtOH and DMSO.

**Time constant**	**EtOH**	**DMSO**
τ_e1_	0.24 ± 0.035 ps	2.7 ± 0.038 ps
τ_e2_	1.2 ± 0.035 ps	>2.5 ns
τ_e3_	>2.5 ns	

The global fitting of **ACyO** in DMSO extracted two time constants, see Table 1. We have assigned the first time constant, τ_e1_ = 2.7 ps, to a collection of processes; geometry relaxation and solvent rearrangement **ACyO** undergoes to reach the S_2_/S_1_ CI (similar to τ_e1_ for **ACyO** in EtOH), followed by vibrational energy transfer along the S_1_ potential energy surface before repopulating the electronic ground state, mediated via the S_1_/S_0_ CI (similar to τ_e2_ for **ACyO** in EtOH). As with **ACyO** in EtOH, the second time constant, τ_e2_ > 2.5 ns, corresponds to any **ACyO** that is still populated in the S_1_ excited state beyond the final time delay of our experiment. Based on the above assignments, we attribute the ESA at ~400 nm which grows in within ~5 ps and persists beyond the final time delay in the TEA spectra of both solvents (Figures 2A,B) to ESA from the S_1_ minimum. For **ACyO** in DMSO, the ESA at extended time delays (2.5 ns) is still relatively large in intensity; however, from our TVA spectra we know that a large percentage of **ACyO** molecules have already returned to their electronic ground state within ~5 ps, see later discussion. Plausible explanations are that the strength of the ESA from the S_1_ minimum is greater than at other locations along the potential energy surface, or the ESA at early time delays is dampened by a competing stimulated emission. Thus, a reduced population on the S_1_ minimum could (and evidently does) result in a more intense ESA. Further to this, we have determined through our TVA spectra that the relaxation mechanism in both solvents is the same, however, there is evidently a solvent dependence on the strength of the ESAs from different positions on the relaxation pathway. For example, the strength of the ESA from the S_1_ minimum in EtOH is evidently weaker than the strength of the ESA from the S_1_ minimum in DMSO. This highlights the influence of solvent on the oscillator strength, which was previously discussed by Woolley et al. (2018). Furthermore, our assignment here (and below) of population trapped on S_1_ concurs with previous work in that there is a barrier to the S_1_/S_0_ CI (Losantos et al., 2017; Woolley et al., 2018).

**Figure 3:**
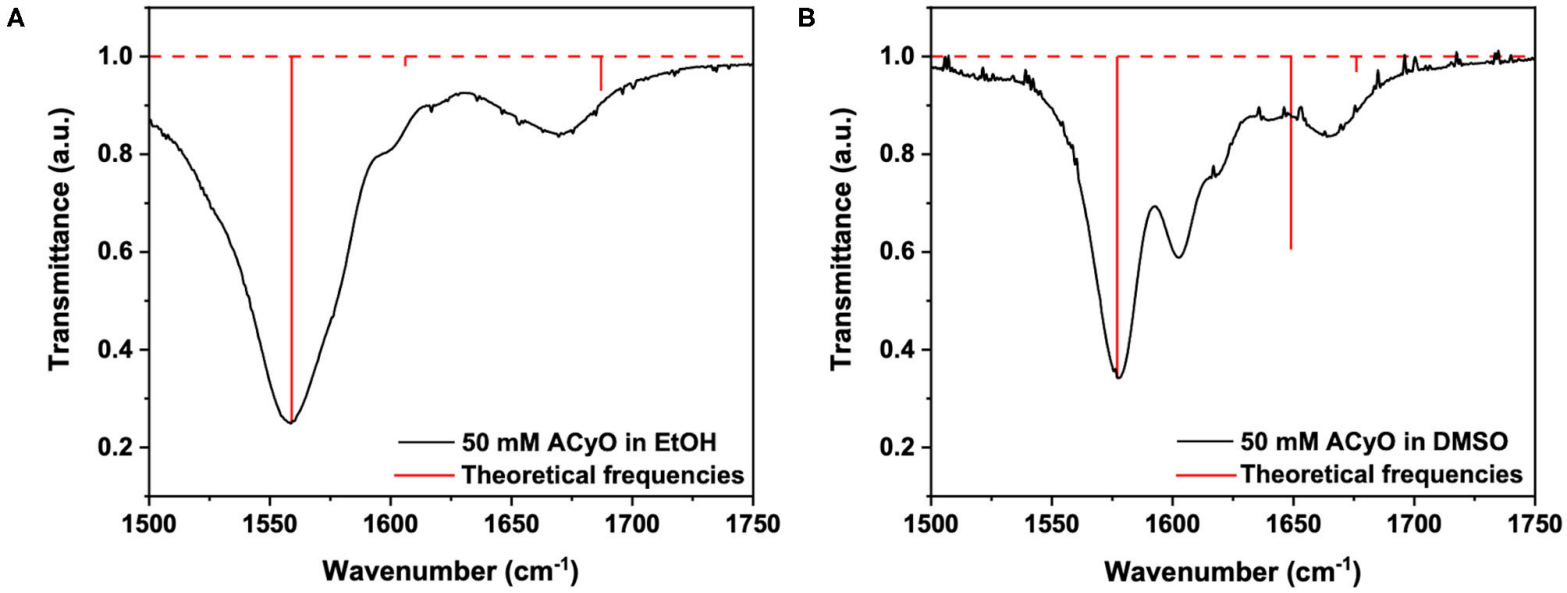
Steady-state FTIR spectra (black lines) of 50 mM **ACyO** in **(A)** EtOH and **(B)** DMSO over 1,500–1,750 cm^−1^. Overlaying the FTIR spectra are the predicted frequencies of 4 averaged explicit-solvent snapshots for each solvent computed at the PBE0/cc-pVTZ level of theory and scaled with the scaling factors of 0.997 and 0.982, respectively, for **ACyO** in EtOH and DMSO (see text for details). The predicted vibrational frequencies are represented by red vertical lines.

However, we highlight again that no indication on the percentage of **ACyO** trapped in the S_1_ excited state can be inferred from the TEA spectra. To glean further insight into this, we conducted both TVAS and steady-state irradiation studies. To begin the TVAS discussion, we must first look at the FTIR spectra of **ACyO** in EtOH and DMSO and assign the vibrational modes to the peaks of interest. Table 2 gives the averaged explicit-solvent calculated S_0_ frequencies (with associated scaling factors of 0.997 and 0.982 for EtOH and DMSO, respectively) and associated vibrational modes. Figure 3 is a visual representation of the calculated frequencies overlaying the FTIR spectra for each **ACyO** solution between 1,500 and 1,750 cm^−1^. Only this region of the FTIR spectra is displayed as it is the region that is not masked by solvent absorption and includes the strongest peaks. The relative strengths of the vibrations have also been scaled to match the reference peaks at 1,559 and 1,577 cm^−1^ for **ACyO** in EtOH and DMSO, respectively.

**Table 2:** Computed S_0_ and S_1_ vibrational frequencies and their associated vibrational modes at the PBE0/cc-pVTZ level of theory for **ACyO** in EtOH and DMSO between 1,500 and 1,750 cm^−1^.

**Solvent**	**S_0_ frequencies, cm^−1^ (Rel. strength)**	**Vibrational mode S_0_**	**S_1_ frequencies, cm^−1^ (Rel. strength)**	**Vibrational mode S_1_**
EtOH	1,559 (298)	C_2_=C_3_ stretch + C_1_=O_7_ stretch + C_2_-H_9_ bend	1,517 (12)	H_10_-C_4_-H_11_ scissor
	1,605 (4)	C_2_=C_3_ stretch + C_1_=O_7_ stretch + H_16_-N_8_-H_17_ scissor	1,565 (46)	C_2_=C_3_ stretch + H_10_-C_4_-H_11_ scissor + N_8_-H_17_ bend + C_2_-H_9_ bend
	1,685 (27)	H_16_-N_8_-H_17_ scissor	1,714 (34)	H_16_-N_8_-H_17_ scissor
DMSO	1,577 (185)	C_2_=C_3_ stretch + C_4_-H_10_ bend + N_8_-H_17_ bend + C_2_-H_9_ bend	1,523 (91)	C_2_=C_3_ stretch + C_4_-H_10_ bend + N_8_-H_17_ bend + C_2_-H_9_ bend
	1,647 (111)	C_1_=O_7_ stretch + H_16_-N_8_-H_17_ scissor	No corresponding frequency	
	1,675 (9)	H_16_-N_8_-H_17_ scissor	1,645 (44)	H_16_-N_8_-H_17_ scissor

*The presented frequencies are an average of four explicit-solvent snapshots except for the S_1_ EtOH frequencies which are an average of two explicit-solvent snapshots. Scaling factors of 0.997 and 0.982 were applied to the calculated frequencies for **ACyO** in EtOH and DMSO, respectively. See text for details*.

The FTIR spectra for **ACyO** in EtOH and DMSO have many differences such as: peak positions; peak widths; peak intensities; and, to a lesser extent, assigned vibrational modes. This highlights how solvent environment effects the observed FTIR spectra and provides a further justification for our choice to implement explicit-solvent calculations. Similarities between the two spectra are that the strongest peak in each spectrum corresponds to the C_2_=C_3_ stretch (given in Table 2 are the additional vibrational modes for this peak in each solvent but for simplicity it is referred to as the C_2_=C_3_ stretch herein) and the weaker peaks at highest wavenumbers in the presented spectra in Figure 3 correspond to the H_16_-N_8_-H_17_ scissor mode, see inset of Figure 1 for atom numbers. A similar pattern was observed in a previous study which computed the vibrational frequencies for **ACyO** in water and acetonitrile adding further credence to our assignment (Sui et al., 2012).

Generally, the calculated peak positions are in good agreement with the experimental data with the largest error being <20 cm^−1^ for the majority of the calculated frequencies. The only exception to this is the calculated frequency of 1,647 cm^−1^ for **ACyO** in DMSO which is shifted ~45 cm^−1^ to a higher wavenumber compared to the experimental peak. This could be explained by the fact we only consider two DMSO molecules interacting with **ACyO** in our calculations compared to a full solvation shell interaction between solute and solvent. However, due to the reasonable accuracy for all other frequencies and because this vibrational mode does not have a corresponding S_1_ frequency (see later discussion), the computational expenditure required for a full solvent shell calculation is deemed unnecessary.

**Figure 4:**
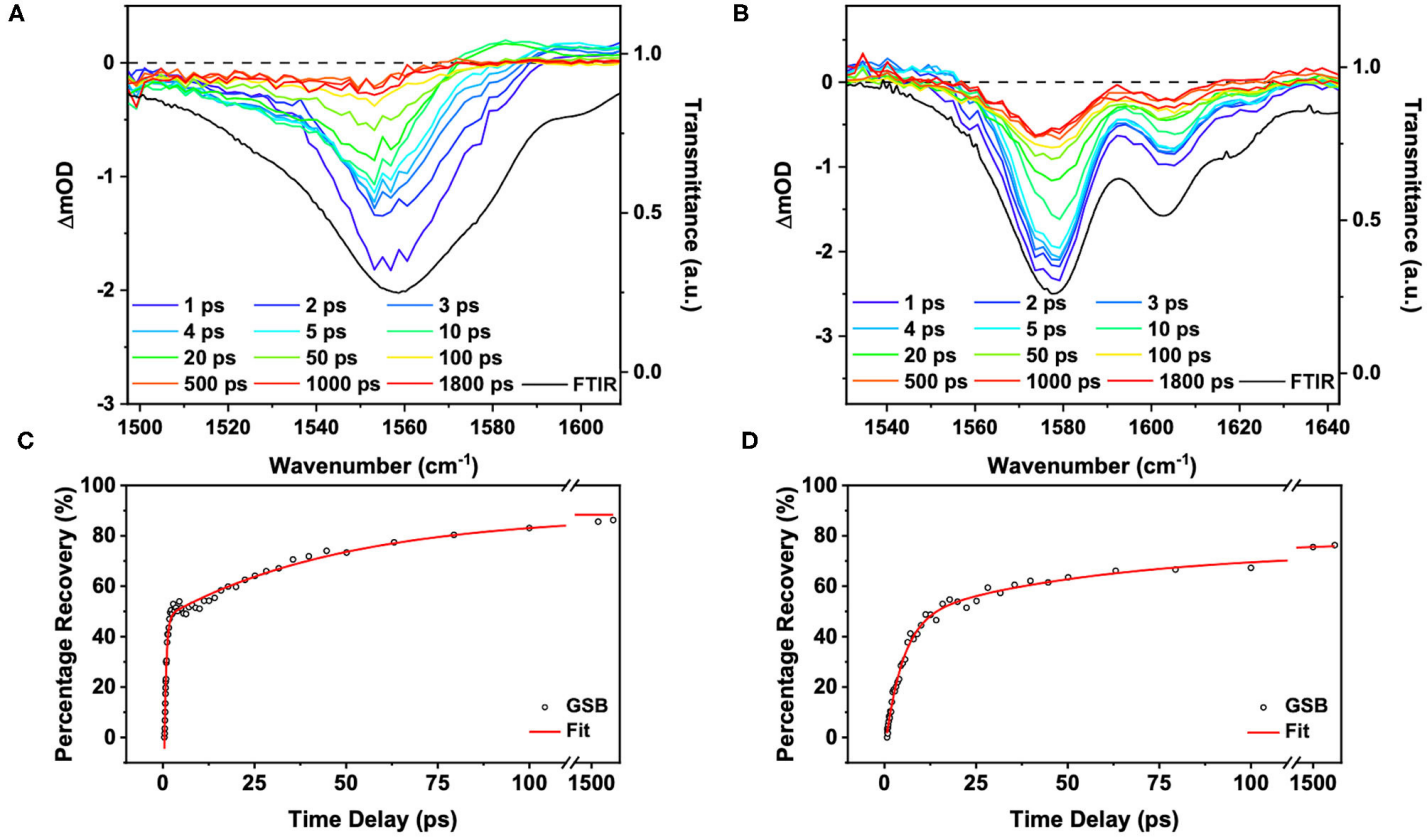
TVA spectra at selected time delays and steady-state FTIR of 50 mM **ACyO** in **(A)** EtOH photoexcited at 285 nm and **(B)** DMSO photoexcited at 280 nm. Scale on left of plots correspond to the change in optical density for the TVA spectra (colored lines) and scale of the right corresponds to the transmittance for the FTIR spectra (black lines). **(C)** Tri-exponential fit of the percentage recovery of the integrated GSB signal from the TVA spectra for **ACyO** in EtOH (1,540–1,550 cm^−1^). **(D)** Tri-exponential fit of the percentage recovery of the integrated GSB signal from the TVA spectra for **ACyO** in DMSO (1,560–1,585 cm^−1^). In both **(C,D)**, the open circles are the integrated GSB signal converted to percentage recovery and the red line is the fit. The time delays are plotted linearly until 110 ps then there is a break until 1,300 ps. Between 1,300 and 1,900 ps the scale is plotted logarithmically to show that the fit does not tend to zero.

The TVA spectra for **ACyO** in EtOH and DMSO and their associated tri-exponential fits for the GSB are shown in Figure 4. Global fitting was not implemented here as we were only interested in the GSB recovery of the probed vibrational modes. Furthermore, we note that our tri-exponential fits begin at the GSB signal maximum; for **ACyO** in EtOH this was 0.4 ps, and for **ACyO** in DMSO this was 0.7 ps. In fitting this way, we avoid any coherent artifacts that occur at early time delays such as perturbed free induction decay (Hamm, 1995). We start the TVAS discussion by first highlighting that in both solvents, full GSB recovery is not achieved at Δ*t* = 1.8 ns. This is in accordance with the TEA spectra *supra* which show excited state dynamics persisting beyond Δ*t* = 2.5 ns. The extracted GSB recovery quantum yields from the integrated signal of each GSB feature were calculated to be 86 and 76% for **ACyO** in EtOH (over 1,540–1,550 cm^−1^, see later discussion for our choice to use this integration region) and DMSO (over 1,560–1,585 cm^−1^) respectively. Greater GSB recovery is observed for **ACyO** in EtOH compared to **ACyO** in DMSO by 10%. A similar trend was observed for the long-term irradiation studies of **ACyO** presented below. The two are intuitively related and imply that more **ACyO** molecules in EtOH are able to overcome the barrier to access the S_1_/S_0_ CI within Δ*t* = 1.8 ns. Therefore, there is less population trapped in the electronic excited state that can undergo pathways leading to photodegradation compared to **ACyO** in DMSO. We note here that the GSB recovery quantum yields we quote only consider GSB recovery that occurs within 1.8 ns, and it is possible that further electronic ground state repopulation may occur after that time.

The time constants extracted from the fit for **ACyO** in EtOH (1,559 cm^−1^ peak) and DMSO (1,577 cm^−1^ peak) are reported in Table 3. We begin by assigning the dynamical processes of the extracted time constants for **ACyO** in EtOH. The first time constant, τ_v1_ = 0.53 ps, is on a similar timescale to τ_e2_ from the TEA spectra fit which would suggest that similar dynamics are occurring as we have reported; geometry relaxation and solvent rearrangement of **ACyO**, followed by vibrational energy transfer on S_1_ and then repopulation of S_0_. However, as TVAS only measures the GSB recovery of the fundamental vibration (being probed) in the electronic ground state, it provides little information about the dynamics in the electronic excited state, making comparison between τ_e2_ and τ_v1_ tentative. As a result, we note that τ_v1_ may only be incorporating some of the electronic excited state dynamics and repopulation of the electronic ground state is most likely occurring on a longer time scale. The second time constant for the TVA spectra, τ_v2_ = 49 ps, is associated with the vibrational cooling of the electronic ground state through vibrational energy transfer, both intramolecular and intermolecular (to solvent). This longer timescale can only be observed in the TVA spectra as the GSB of **ACyO** falls outside of the probe window for our TEAS experiment. As a result, no spectral signatures of vibrational cooling on the S_0_ are present in the TEA spectra, and thus no time constant can be assigned to this photophysical process. Furthermore, we do not observe a blue-shifting of the transient vibrational bands with time that is often associated with the anharmonicity of vibrations (Nibbering et al., 2005), and instead observe a slight red-shift for **ACyO** in EtOH (Figure 4A). We reconcile this through the following: firstly, it is possible that the probed vibration is relatively harmonic; and secondly, the ESA from the S_1_ minimum between ~1,560 and 1,570 cm^−1^, which contributes toward the GSB recovery and causes an asymmetric line shape at extended time delays (see later discussion), is likely the main cause of the apparent red-shift. For **ACyO** in DMSO (Figure 4B), neither a red-shift or a blue-shift is observed, providing confidence that the probed vibrations are relatively harmonic. The final time constant for **ACyO** in EtOH, τ_v3_ > 1.8ns, is due to the incomplete GSB recovery. Drawing reference to our TEAS data, this is very likely attributed to the long-lived **ACyO** trapped in the minimum of the S_1_.

**Table 3:** Time constants and associated errors extracted from the tri-exponential fit of the integrated GSB signal from the TVA spectra for **ACyO** in EtOH (1,540–1,550 cm^−1^) and DMSO (1,560–1,585 cm^−1^).

**Time constant**	**EtOH (1,559 cm^−1^)**	**DMSO (1,577 cm^−1^)**
τ_v1_	0.53 ± 0.028 ps	4.7 ± 0.35 ps
τ_v2_	49 ± 2.9 ps	55 ± 9.0 ps
τ_v3_	>1.8 ns	>1.8 ns

The calculated S_1_ frequencies and associated vibrational modes for **ACyO** in EtOH can be found in Table 2 and were scaled using the same scaling factor that was applied to the S_0_ frequencies, 0.997. The reported frequencies are an average of two snapshots due to difficulties experienced when running our EtOH explicit-solvent excited state calculations, see the Supplementary Material for more details. We draw confidence on the validity of these results from the similarity between the two snapshots, alongside the consistency of the results observed in DMSO. Future studies may invest further into converging more snapshots, but this has a high computational cost and is beyond the scope of the work. The only S_1_ frequency which had a clear corresponding S_0_ frequency between 1,500 and 1,750 cm^−1^ for **ACyO** in EtOH was for the H_16_-N_8_-H_17_ scissor vibrational mode. This mode is shifted ~30 cm^−1^ in the S_1_ to a higher wavenumber of 1,714 cm^−1^. Additionally, two other excited state frequencies were found during the calculation in the region of interest. These were computed as 1,517 and 1,565 cm^−1^ and they correspond to the H_10_-C_4_-H_11_ scissor and the C_2_=C_3_ stretch (plus several other vibrational modes), respectively, see Table 2 for full vibrational mode assignment. These vibrational modes are convoluted on top of the GSB of the C_2_=C_3_ stretch at 1559 cm^−1^ that we probed in the TVAS experiment. The relative strengths of the two S_1_ frequencies are significantly smaller than the S_0_ frequency therefore, we would expect any ESAs to be much weaker in intensity compared to the GSB. That being said, from the TVA spectra in Figure 4, a small ESA feature is observable at a wavenumber of ~1,570 cm^−1^ and above; present between 10–20 ps and decaying by 50 ps. We are unable to definitely pinpoint the origin of this feature. Possible suggestions include probing: (1) the C_2_=C_3_ stretch in S_1_; and (2) a higher lying vibration in **ACyO** in the ground electronic state which subsequently cools. Indeed, the decay of this feature matches τ_v2_ adding credence to the latter assignment. Importantly, at 1.8 ns, the asymmetry of the GSB feature insinuates that an ESA is contributing toward the GSB recovery between ~1,560 and 1,570 cm^−1^. This matches the computed S_1_ frequency at 1,565 cm^−1^, therefore, we assign it to the population of **ACyO** trapped in the S_1_ minimum. The ESA does not emerge above baseline due to the fact the transition is six times weaker than the probed electronic ground state transition, see Table 2. Due to the convolution of peaks, we highlight that for the GSB recovery quantum yield and fitting of the GSB, a smaller region (1,540–1,550 cm^−1^) was integrated between to negate as many contributions from the overlapping ESAs as possible. We appreciate that there is still a possibility that the ESA is contributing to the GSB recovery, hence we report the GSB recovery quantum yield of 86% as being the upper limit in this case.

The TVA spectra of **ACyO** in DMSO is more straightforward to interpret due to no convoluted S_1_ frequencies computed by TDDFT calculations. A tri-exponential fit of the integrated signal of the 1,577 cm^−1^ vibration (1,560–1,585 cm^−1^) can be found in Figure 4 and its corresponding time constants can be found in Table 3. The same assignment of the time constants is made for **ACyO** in DMSO as that reported above for **ACyO** in EtOH. τ_v1_ (4.7 ps) is assigned to the geometry relaxation and solvent rearrangement **ACyO** undergoes to access the S_2_/S_1_ CI, followed by vibrational cooling on the S_1_ and traversing through the S_1_/S_0_ CI to repopulate the electronic ground state. Like in **ACyO** in EtOH, the timescales of τ_e1_ and τ_v1_ are similar. τ_v2_ (55 ps) is assigned to the vibrational cooling of **ACyO** on the S_0_ through vibrational energy transfer. Again, comparison to the TEA spectra cannot be made for τ_v2_ due to TEAS experimental limitations as discussed above. The final time constant, τ_v3_ > 1.8 ns, is assigned to any **ACyO** molecules trapped in the minimum of the S_1_ potential energy surface.

We conclude the TVAS discussion by evaluating the S_1_ vibrational frequency calculations for **ACyO** in DMSO which can be found in Table 2. These computed frequencies are an average of four explicit-solvent snapshots and were simpler to assign to their corresponding electronic ground state frequencies, unlike **ACyO** in EtOH. For the C_2_=C_3_ stretch vibration, calculated frequency values of 1,577 cm^−1^ in the S_0_ state and 1,523 cm^−1^ in the S_1_ state are reported in Table 2. For the H_16_-N_8_-H_17_ scissor vibration, the calculated frequency values are 1,675 cm^−1^ in the S_0_ state and 1,645 cm^−1^ in the S_1_ state. Therefore, upon S_1_ ← S_0_ excitation, the harmonic frequencies of both vibrations are shifted to a lower wavenumber; the frequency of the C_2_=C_3_ stretch vibration by ~50 cm^−1^ and the frequency of the H_16_-N_8_-H_17_ scissor vibration by ~30 cm^−1^. There was no corresponding S_1_ frequency for the C_1_=O_7_ stretch plus H_16_-N_8_-H_17_ scissor that was computed as 1,647 cm^−1^ in the electronic ground state. Additionally, there were no other new S_1_ frequencies appearing between 1,500 and 1,750 cm^−1^ and this insinuates that no ESA features should be present in the TVA spectra, which is the case as seen in Figure 4B. As a result, we are confident that the GSB recovery quantum yield for **ACyO** in DMSO (76%) is a more accurate value than the GSB recovery quantum yield for **ACyO** in EtOH (86%). We add that a degenerate pump-probe experiment would complement the observed GSB recovery quantum yields obtained from our TVAS experiments; however, for the present study, the TVAS data provides a good starting point with which to progress with future studies.

**Figure 5:**
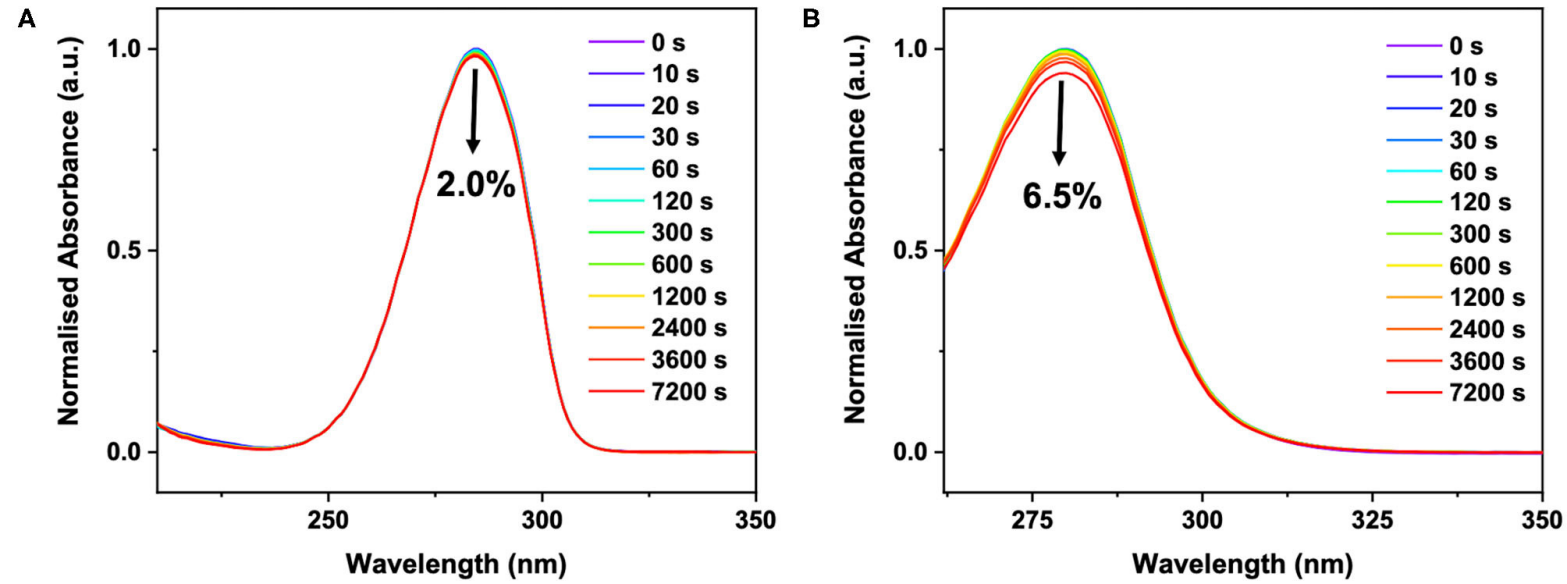
UV/visible spectra of ~40 μM **ACyO** in **(A)** EtOH and **(B)** DMSO taken over varying durations of irradiation in a solar simulator. Black arrow on each plot denotes a decrease in absorbance at the absorption maxima after 7,200 s and corresponds to 2.0% for **ACyO** in EtOH and 6.5% for **ACyO** in DMSO.

In our efforts to link our ultrafast dynamics studies of **ACyO** (and the GSB recovery quantum yields thereof) to the long-term photostability of **ACyO**, we performed long-term irradiation studies and the results are presented in Figure 5. The data shows that after 7,200 s, only 2.0 and 6.5% degradation of signal at the absorption maxima occurred for **ACyO** in EtOH and DMSO, respectively. Control measurements confirmed that all degradation observed was due to photodegradation, see Supplementary Figure 8. The impressive levels of photostability of **ACyO** in both solvents must stem from highly efficient repopulation of the electronic ground state as we observed in our TVAS studies *supra*. In the long-term irradiation study, we see no emergence of an absorbance at ~400 nm in either solvent, eliminating the possibility that the long-lived feature in the TEA spectra is a photoproduct. This provides further support that the long-lived feature observed in our TEA and TVA spectra is **ACyO** population trapped in S_1_ state. Losantos et al. (2017) predicted that the cyclohexenone unit they studied would radiatively decay after becoming trapped on the S_1_ in order to repopulate the molecules' electronic ground state. However, in accordance with Woolley et al. (2018) we observed no radiative decay implying that **ACyO** must recover to the electronic ground state non-radiatively. We draw this conclusion from the fact that no stimulated emission features (induced by one-photon absorption of the pump) are present in the TEA spectra for **ACyO** in both EtOH and DMSO, and that no emission was observed in the steady-state emission spectra of **ACyO** in both solvents when compared to blank scans of solvent-only solutions (spectra not shown). The lack of observed fluorescence would suggest that there is very poor Franck-Condon overlap between the S_1_ minimum and the electronic ground state. These steady-state results further support our conclusion that the S_1_/S_0_ CI is accessed by >75% of **ACyO** molecules on ultrafast timescales as they are able to overcome the mild barrier from the S_1_ minimum (0.04 eV) (Woolley et al., 2018).

For the **ACyO** population remaining in the excited state at extended time delays, >1.8 ns, it is likely that further repopulation of the electronic ground state and some photoproduct formation occurs. It is important to highlight that the systems we have studied are simple, isolated systems, therefore, we have observed repopulation of the electronic ground state at time beyond 1.8 ns. However, in a more complex environment like a sunscreen formulation, the population of **ACyO** in the S_1_ excited state persisting beyond 1.8 ns has a greater chance of either forming a photoproduct or transferring that energy through collisions. This reinforces the importance of why an ideal UV filter would return to its electronic ground state on ultrafast timescales and why we conclude that **ACyO** is not a perfect UV filter candidate. We close the discussion by reiterating that more photoproduct formation appears to be occurring for **ACyO** in DMSO which is intuitively related to the lower GSB recovery quantum yield extracted from the TVA spectra presented above. The greater population persisting in the S_1_ excited state at extended time delays, >1.8 ns, increases the probability for photoproduct formation.

## Conclusion

Throughout the present work we have demonstrated that although **ACyO** has a long-lived excited state that persists beyond 2.5 ns, the extent of ground state recovery on ultrafast timescales is relatively high. We have determined that >75% of photoexcited **ACyO** in EtOH and DMSO is returning to its electronic ground state within 1.8 ns. These numbers appear to corroborate why **ACyO** displays good photostability in the isolated systems studied. Upon photoexcitation, we demonstrate that the majority of **ACyO** molecules have sufficient energy to overcome the S_1_/S_0_ CI barrier and relax non-radiatively on ultrafast timescales. Although **ACyO** displays long-lived features in accordance with previous studies (Losantos et al., 2017; Woolley et al., 2018), we propose that the cyclohexenone core is not as poor a UV filter as initially predicted, supporting nature's choice of the cyclohexenone core in MAAs. However, for an ideal UV filter we would ideally like as-close-to complete GSB recovery within ultrafast timescales; further studies on molecules with a cyclohexenone unit are therefore most-certainly warranted so as to provide clearer insight in to their photoprotective mechanisms, and how these mechanisms are influenced with substituent. In particular, how the GSB recovery quantum yield is influenced as we move progressively toward gadusol is one avenue to be explored, which could eventually guide the future design of UV filters based on MAAs.

In this present work, we have demonstrated that TVAS is a very powerful method when allied with appropriate high-level theory (in this case using explicit-solvent methodology which is important to consider when solute and solvent interact strongly with each other) for extracting accurate GSB recovery information. The combination of this high-level theory and TVAS enables vibrational ESA bands to be identified when selecting an integration region, allowing for the origins of a molecule's photostability to be traced. TVAS provides invaluable dynamical information that cannot be extracted from TEAS. Therefore, we suspect that this technique will see increasing use in the field of sunscreen science.

## Data Availability Statement

The datasets presented in this study can be found in online repositories. The names of the repository/repositories and accession number(s) can be found below: Zenodo repository doi: 10.5281/zenodo.3885450.

## Author Contributions

ALW conducted the majority of the experimental and computational work and took lead in writing the manuscript. MAPT ran the molecular dynamics simulations and provided support for the computational analysis. MAPT and JMW supervised the writing of the manuscript. DJLC and MDH helped conduct the transient vibrational absorption spectroscopy experiments presented in this paper. VGS conceived the experiment, supervised the writing of the manuscript, and supported the data analysis. All authors contributed to the article and approved the submitted version.

## Conflict of Interest

The authors declare that the research was conducted in the absence of any commercial or financial relationships that could be construed as a potential conflict of interest.

## Abbreviations

UV: ultraviolet
MAA: mycosporine-like amino acid
ACyO: 3-aminocyclohex-2-en-1-one
TEAS: transient electronic absorption spectroscopy
CI: conical intersection
ESA: excited state absorption
GSB: ground state bleach
TVAS: transient vibrational absorption spectroscopy
DFT: density functional theory
EtOH: ethanol
DMSO: dimethyl sulfoxide
TDDFT: time-dependent density functional theory
FTIR: Fourier transform infrared
TEA: transient electronic absorption
MCT: mercury cadmium telluride
TVA: transient vibrational absorption.
