# Supplementary material for: Reinvestigating the Photoprotection Properties of a Mycosporine Amino Acid Motif
Source: Front Chem. 2020 Sep 25;8:574038. doi: 10.3389/fchem.2020.574038 (PMC7546825; doi:10.3389/fchem.2020.574038)
Supplement: Supplementary file 1 [file Data_Sheet_2.PDF]

Supplementary Material

1 List of files included in online Zenodo repository

The Zenodo archive at DOI: 10.5281/zenodo.3885450 contains the underlying data for this publication. This includes:

- Input, geometry and output files for the S_0 and S_1 geometry optimisations, S_0 and S_1 frequency calculations and vertical excitations for **ACyO** in EtOH and DMSO using implicit-solvent models.
- Input, geometry and output files for the S_0 and S_1 geometry optimisations, S_0 and S_1 frequency calculations and vertical excitations for **ACyO** in EtOH and DMSO using explicit-solvent models. Calculations were performed on four snapshots for each solvent.
- Averaged TEAS scans for 4 mM **ACyO** in EtOH and DMSO after chirp correction.
- Averaged TVAS scans for 50 mM **ACyO** in EtOH and DMSO.

2 Transient absorption spectra power dependencies, evolution associated difference spectra, residuals associated with the global fit, and instrument response functions

Power dependency studies were carried out by varying the Topas-Prime output power to several powers around the power used for transient scans. The signal was integrated over 10 nm windows for the transient electronic absorption (TEA) spectra and over ~20 cm^{-1} windows for the transient vibrational absorption (TVA) spectra at a specified pump-probe time delay. The integration window and pump-probe time delay were selected to cover a region of the TEA/TVA spectra that displayed either a positive or negative feature. In a Log(Power) vs Log(Signal) plot, a gradient of ~1 indicates that the observed dynamics are one-photon initiated dynamics. This was the case for the ESAs and GSBs in the TEA and TVA spectra, see **Supplementary Figure 1** and **4**. The negative feature at early time delays in the TEA spectra for **ACyO** in EtOH returned a gradient of ~2 which indicates two-photon initiated dynamics (**Supplementary Figure 1**). As we are only interested in one-photon initiated dynamics, we omitted the negative feature in our global fit of **ACyO** in EtOH.

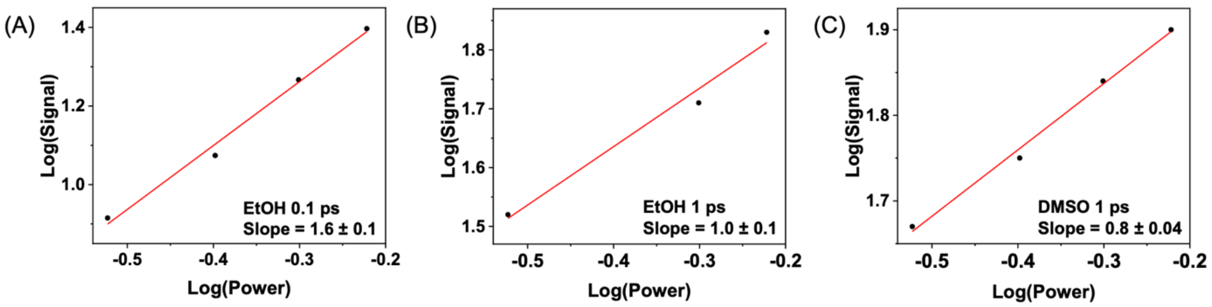

Supplementary Figure 1. Power dependencies for TEA spectra of **ACyO** in **(A)** EtOH, photoexcited at 285 nm. 350 to 360 nm was chosen as the integration window and the pump-probe time delay was 0.1 ps. **(B)** EtOH, photoexcited at 285 nm and **(C)** DMSO, photoexcited at 280 nm. In both **(B)** and **(C)**, 335 to 345 nm was chosen as the integration window and the pump-probe time delay was 1 ps.

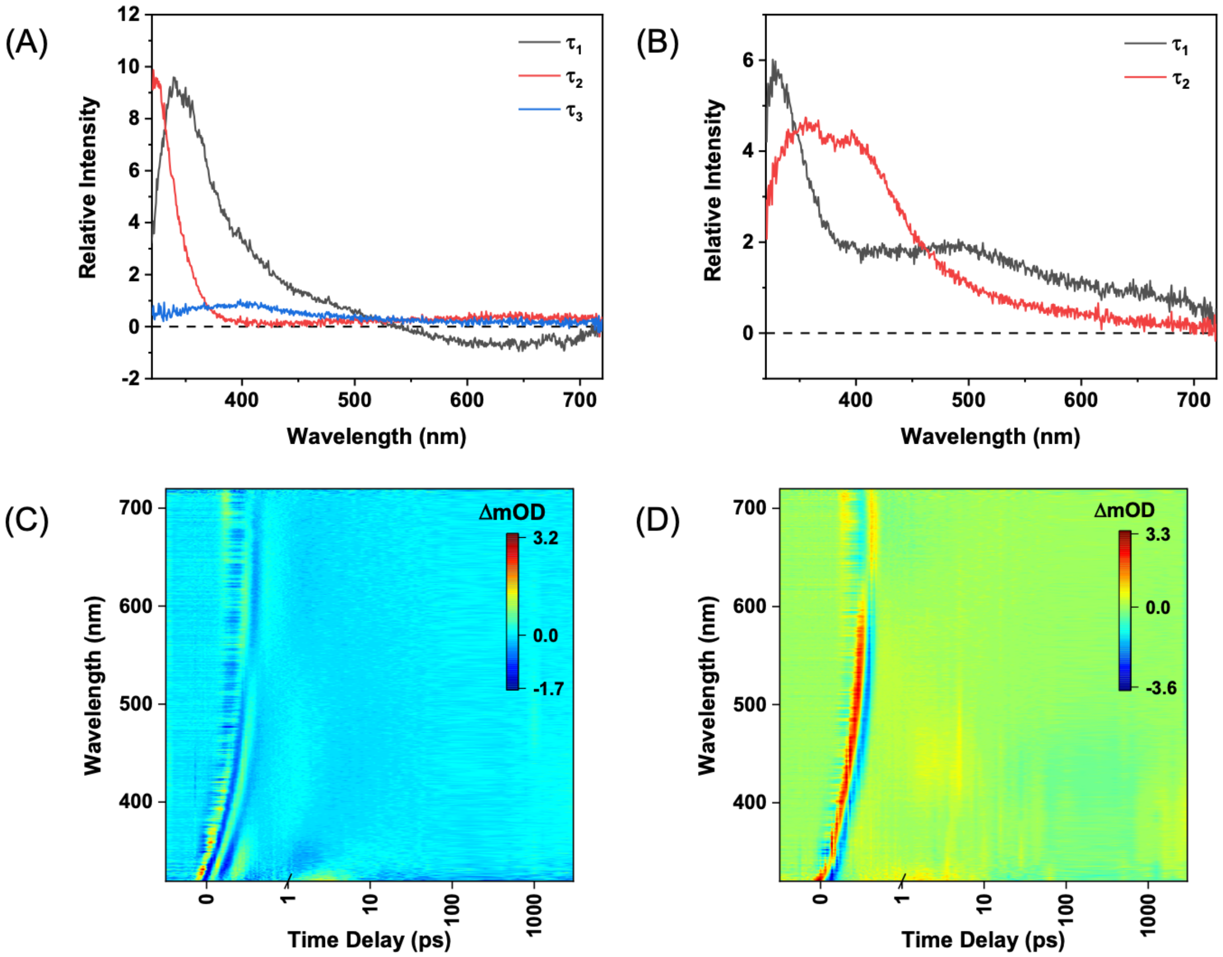

Supplementary Figure 2. Evolution associated difference spectra from the global fitting of **ACyO** in **(A)** EtOH and **(B)** DMSO. The kinetic model used to fit the data is reported in the Materials and Methods section of the main paper. False colour maps of the residuals from the global fitting of **ACyO** in **(C)** EtOH and **(D)** DMSO that have not been chirp corrected. For **(C)** and **(D)**, the time delays are plotted linearly until 1 ps and then as a log scale from 1 to 2500 ps.

The instrument response function, accounting for the temporal resolution of our TEAS measurements, was calculated by fitting a Gaussian over the time-zero artefacts of solvent-only scans and taking the full width half maximum (FWHM). The instrument response functions for the present work are displayed in **Supplementary Figure 3**.

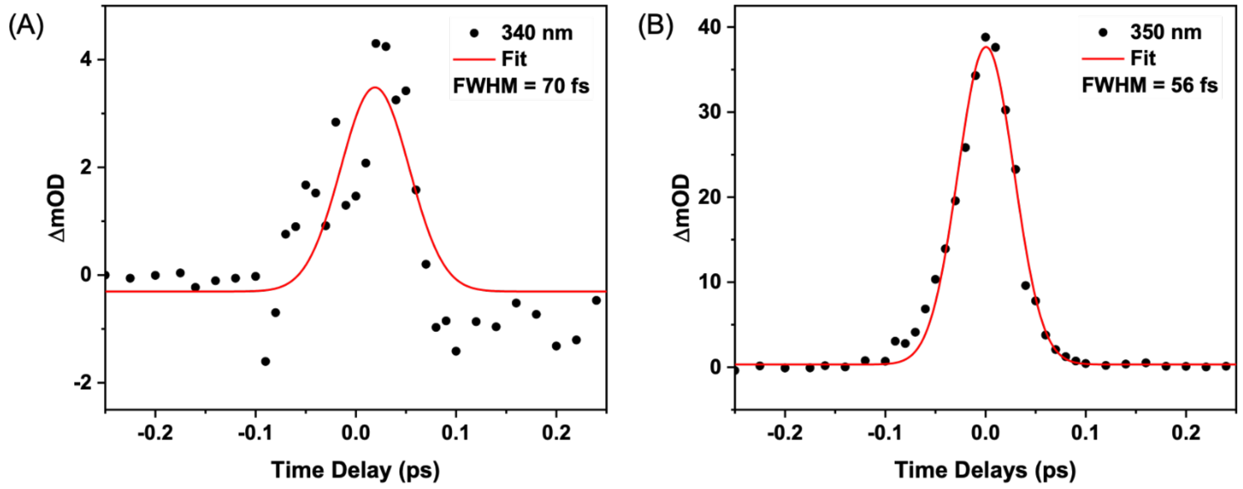

Supplementary Figure 3. Gaussian fit for TEA spectra of **(A)** EtOH only scan (pumped at 285 nm) at a probe wavelength of 340 nm; the returned FWHM is 70 fs, and **(B)** DMSO only scan (pumped at 280 nm) at a probe wavelength of 350 nm; the returned FWHM is 56 fs. The filled black circles are the raw data and the red lines are the gaussian fits.

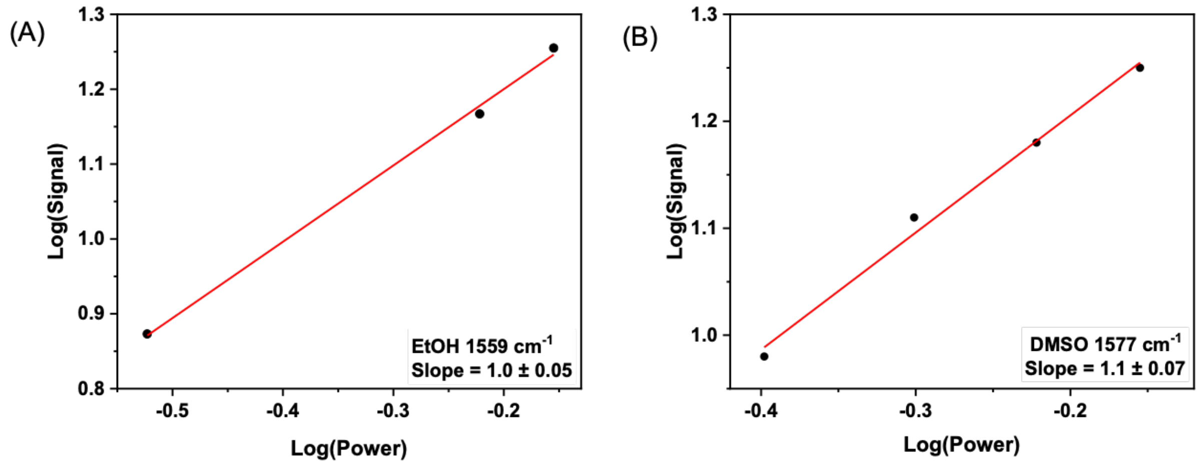

Supplementary Figure 4. Power dependencies for TVA spectra of **ACyO** in **(A)** EtOH photoexcited at 285 nm. 1549 to 1568 cm^{-1} was chosen as the integration window and the pump-probe time delay was 1 ps. **(B)** DMSO photoexcited at 280 nm. 1568 to 1585 cm^{-1} was chosen as the integration window and the pump-probe time delay was 0.7 ps.

3 Implicit-solvent computed vibrational frequencies

The results for the S_0 and S_1 vibrational frequencies, computed with implicit-solvent, between 1500 and 1750 cm^{-1} are given below in **Supplementary Table 1**. **Supplementary Figure 5** is a visual representation of the computed electronic ground state frequencies of **ACyO** in both EtOH and DMSO overlaying their experimental FTIR spectra. As EtOH and DMSO interact strongly with **ACyO**, it was decided that explicit-solvent contributions should be considered. We note that the results for both implicit- and explicit-solvent environments were not perfect, however, our reasons for reporting explicit-solvent results in the main paper are the following. Firstly, in the EtOH calculated S_0 frequencies, the mode at 1639 cm^{-1} is significantly shifted to a lower wavenumber from the experimental peak. Secondly, the assigned vibrational modes for DMSO had discrepancies with previous density functional theory (DFT) calculations and the EtOH calculations conducted in the present work, which all found that the peak at the highest wavenumber in the region of interest corresponds to the H_{16}-N_8-H_{17} scissor mode only (Sui et al., 2012). It is noted that previous studies were conducted in different solvents, however, similar patterns in the vibrational mode assignments can be made. On running explicit-solvent calculations, the DMSO vibrational mode assignments better correlated with patterns found in previous findings and the EtOH calculations from the present work (Sui et al., 2012). Additionally, DMSO explicit-solvent calculations improved the relative intensities of the computed frequencies to better match experimental results. Finally, we highlight that the EtOH explicit-solvent computed S_1 frequencies match the asymmetry that we observe in our TVA spectra at 1.8 ns, see **Figure 4A** of the main paper. Whereas, the computed S_1 frequencies using EtOH implicit-solvent does not provide such an explanation for the observed asymmetry. Also, we note that whether an implicit- or explicit-solvent model is used for **ACyO** in DMSO, the computed S_1 frequencies do not overlap with the peaks probed in the TVAS experiment.

Supplementary Table 1. Computed S_0 and S_1 vibrational frequencies and their associated vibrational modes at the PBE0/cc-pVTZ level of theory for **ACyO** in EtOH and DMSO between 1500 and 1750 cm^{-1} using the COSMO solvent model. A scaling factor of 0.976 was applied to the calculated frequencies for **ACyO** in EtOH and DMSO. The arrows showing the vectors illustrating the vibrational modes of **ACyO** have been scaled with a scaling factor of 2 for clarity.

**Solvent**	**S_0 frequencies, cm^{-1} (Rel. strength)**	**Vibrational mode S_0**	**S_1 frequencies, cm^{-1} (Rel. strength)**	**Vibrational mode S_1**
EtOH	1559 (211)	C_2=C_3 stretch + C_4-H_{10} bend + N_8-H_{17} bend + C_2-H_9 bend 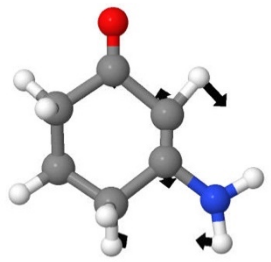	1514 (36)	H_{10}-C_4-H_{11} scissor 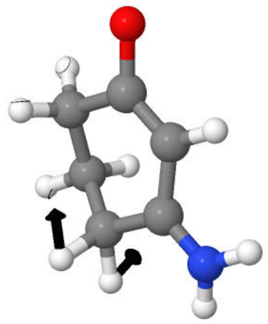

	1597 (46)	$C_1=O_7$ stretch + H_{16}-N_8-H_{17} scissor<br>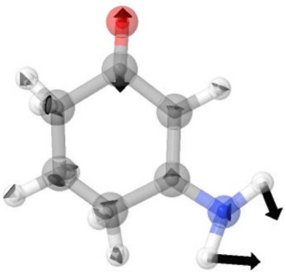	1544 (55)	H_{10}-C_4-H_{11} scissor<br>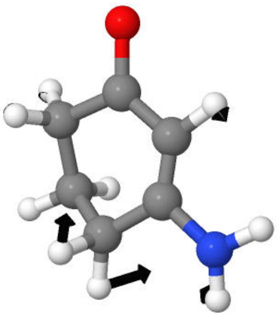
	1639 (17)	H_{16}-N_8-H_{17} scissor<br>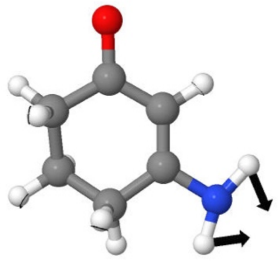	1728 (34)	H_{16}-N_8-H_{17} scissor<br>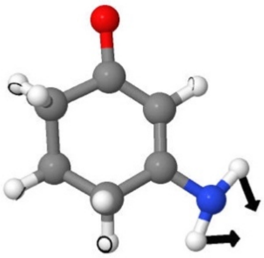
DMSO	1577 (112)	$C_2=C_3$ stretch + C_4-H_{10} bend + N_8-H_{17} bend + C_2-H_9 bend<br>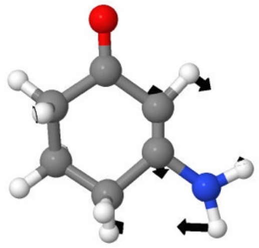	1501 (38)	H_{10}-C_4-H_{11} scissor<br>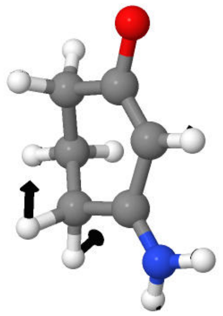
	1620 (107)	H_{16}-N_8-H_{17} scissor<br>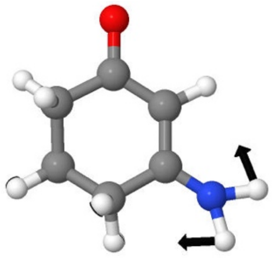	1534 (35)	H_{10}-C_4-H_{11} scissor<br>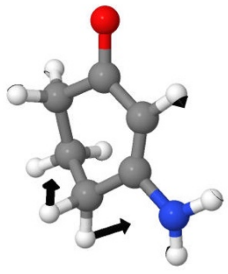
	1659 (35)	$C_1=O_7$ stretch + H_{16}-N_8-H_{17} scissor<br>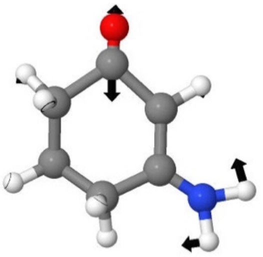	1720 (27)	H_{16}-N_8-H_{17} scissor<br>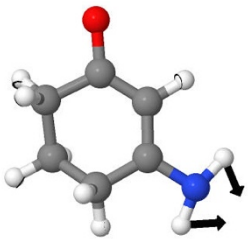

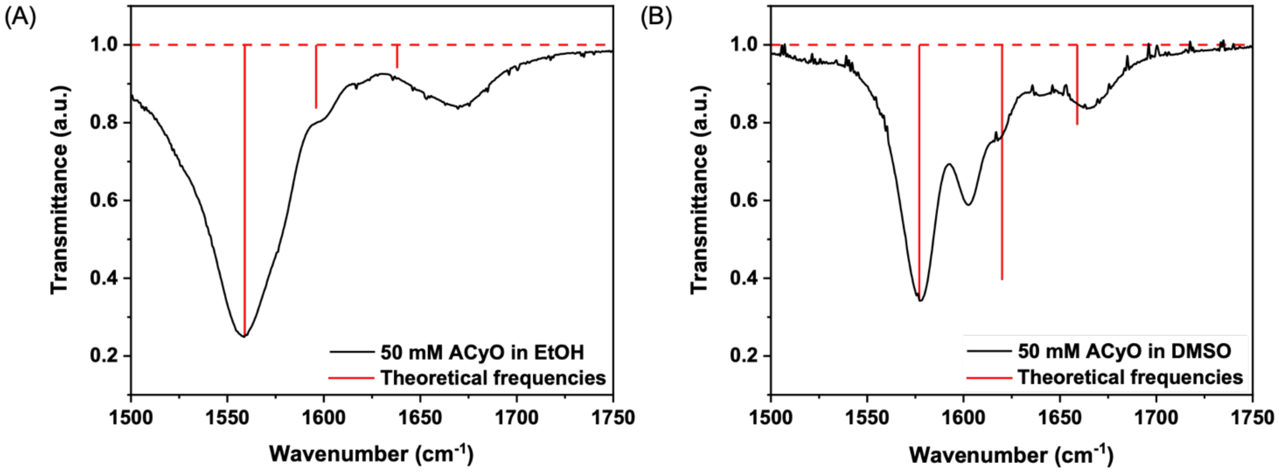

Supplementary Figure 5. Steady-state FTIR spectra (black lines) of 50 mM **ACyO** in **(A)** EtOH and **(B)** DMSO over 1500 to 1750 cm^{-1}. Overlaying the FTIR spectra are the predicted implicit-solvent frequencies computed at the PBE0/cc-pVTZ level of theory and scaled with a scaling factor of 0.976 for **ACyO** in EtOH and DMSO. The predicted vibrational frequencies are represented by red vertical lines.

4 Explicit-solvent geometry optimisation structures and their energy

Supplementary Table 2. Starting explicit-solvent snapshots and their corresponding S_0 and S_1 relaxed geometry computed at the PBE0/cc-pVTZ level of theory. Also reported are the relative energies of the S_1 relaxed geometries with respect to their S_0 relaxed geometries. Geometry relaxation in the S_1 for the EtOH 2 snapshot did not converge at the PBE0/cc-pVTZ level of theory, therefore no S_1 relaxed structure is presented here.

**Before geometry optimisation**	**S_0 relaxed geometry and relative energy**	**S_1 relaxed geometry and relative energy**
EtOH 1<br>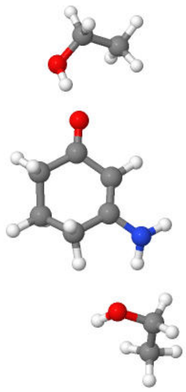	0 eV<br>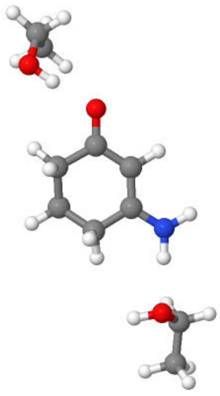	4.5713414 eV<br>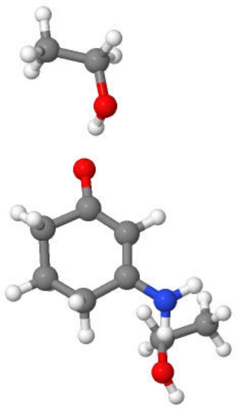

EtOH 2<br>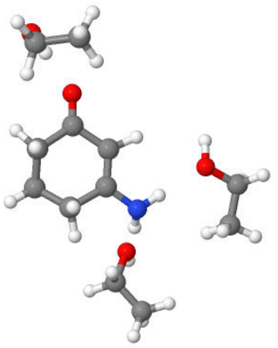	0 eV<br>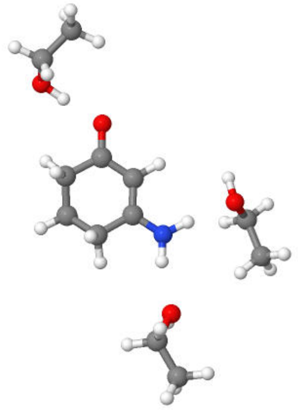	
EtOH 3<br>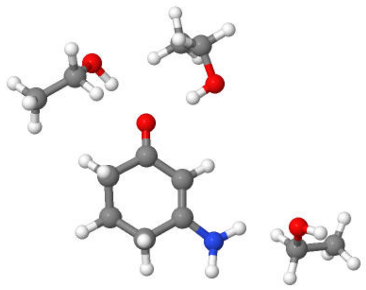	0 eV<br>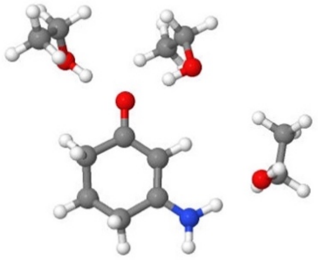	4.6620223 eV<br>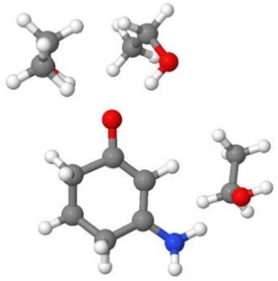
EtOH 4<br>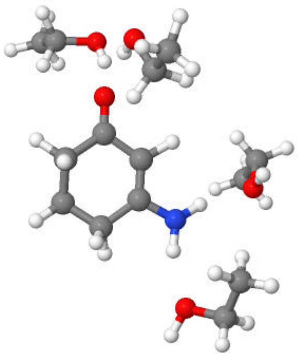	0 eV<br>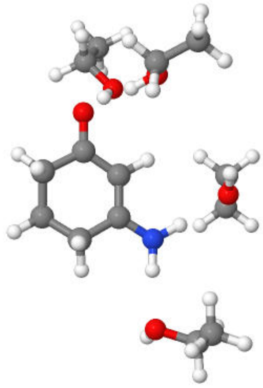	4.5552693 eV<br>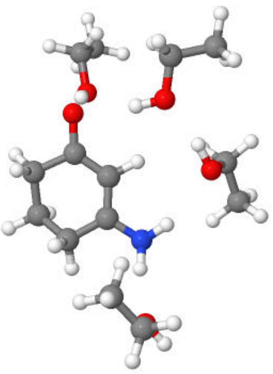
DMSO 1<br>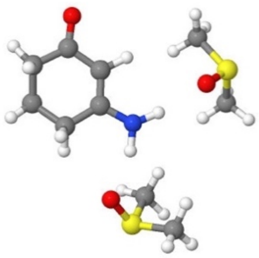	0 eV<br>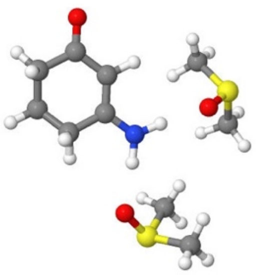	4.1588542 eV<br>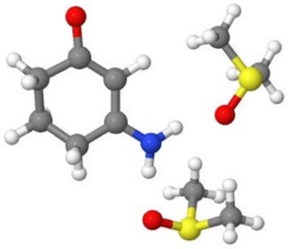

DMSO 2<br>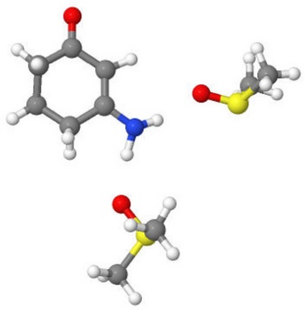	0 eV<br>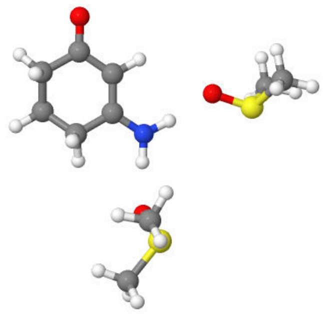	4.1993638 eV<br>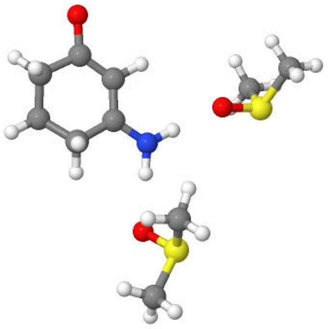
DMSO 3<br>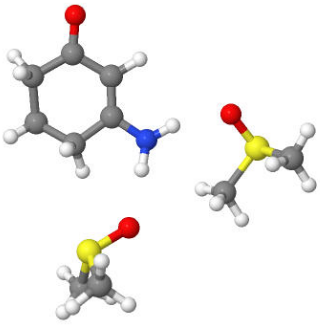	0 eV<br>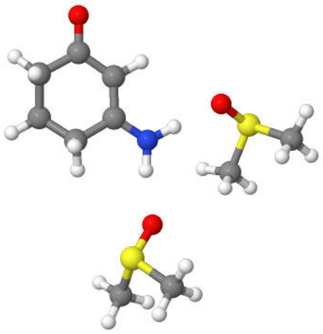	4.1910744 eV<br>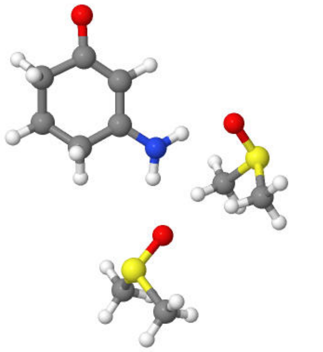
DMSO 4<br>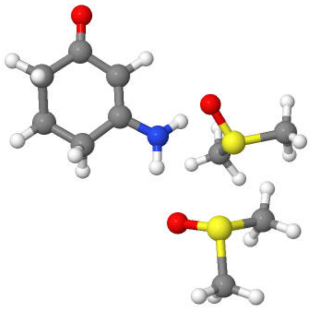	0 eV<br>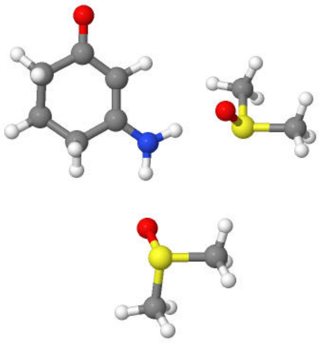	4.1561616 eV<br>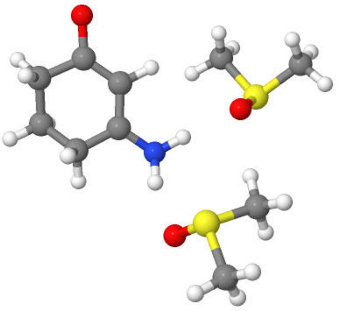

5 Explicit-solvent computed vibrational frequencies

Both EtOH 2 and EtOH 3 S_1 frequencies were not included in the averaged S_1 frequencies presented in **Table 2** of the main paper. For the EtOH 2 snapshot, excited-state geometry relaxation along the S_1 energy surface did not converge at the PBE0/cc-pVTZ level of theory. Multiple pre-optimisation calculations were ran to aid convergence however, we believe that there was an inconsistency between the predicted forces acting upon the molecule in the excited state and the corresponding predicted energy surface. Consequently, it appears as if the relative energy of the structure increased as the optimisation continued and forces reduced. This issue could potentially be solved by using a larger basis set, however this work was already computationally expensive and this would further increase that. As a result, no S_1 frequencies were calculated for the EtOH 2 snapshot. In the case of the EtOH 3 snapshot, the S_1 geometry relaxation did converge without complications but the calculated S_1 frequencies and vibrational modes do not match those of the EtOH 1 and EtOH 4 snapshots. Additionally, of the four EtOH environments studied, EtOH 3 was the least observed in all 200 generated snapshots. Therefore, we can assume this EtOH environment is an outlier and so it was ignored when averaging the S_1 frequencies.

We draw confidence from the similarity between the two EtOH snapshots, as well as the similarity of results for the four snapshots in DMSO, that averaging the S_1 frequencies for the two EtOH snapshots is sufficient. Following these results, we conclude that explicit-solvent EtOH calculations in the excited state can be difficult to work with and require significant computational expenditure. As a result, caution will be taken when conducting similar calculations in the future.

Supplementary Table 3. Computed S_0 and S_1 vibrational frequencies and their associated vibrational modes at the PBE0/cc-pVTZ level of theory for **ACyO** in EtOH and DMSO between 1500 and 1750 cm^{-1}. The presented frequencies are for 8 explicit-solvent snapshots extracted from molecular dynamics simulations and the scaling factors applied to each explicit-solvent snapshot are reported in the table below. All frequencies except for EtOH 3 S_1 frequencies were averaged to give the reported frequencies in **Table 2** of the main paper. The arrows showing the vectors illustrating the vibrational modes of **ACyO** have been scaled with a scaling factor of 3 for clarity.

**Solvent (Scaling factor)**	**S_0 frequencies cm^{-1} (Rel. strength)**	**Vibrational mode S_0**	**S_1 frequencies cm^{-1} (Rel. strength)**	**Vibrational mode S_1**
EtOH 1 (0.991)	1559 (277)	C_2=C_3 stretch + C_1=O_7 stretch + C_2-H_9 bend 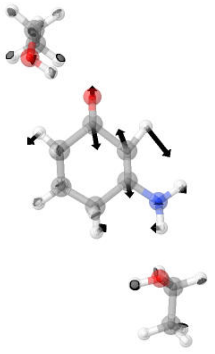	1524 (14)	H_{10}-C_4-H_{11} scissor 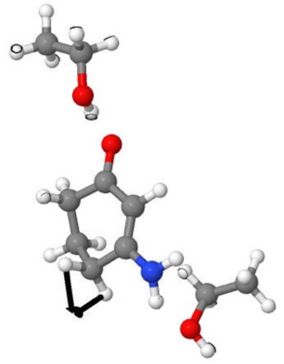
	1598 (3)	C_2=C_3 stretch + C_1=O_7 stretch + H_{16}-N_8-H_{17} scissor 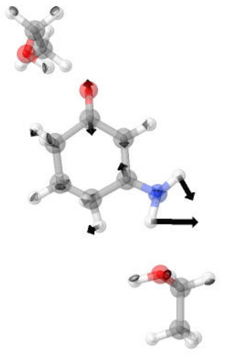	1579 (47)	C_2=C_3 stretch + C_4-H_{10} bend + N_8-H_{17} bend + C_2-H_9 bend 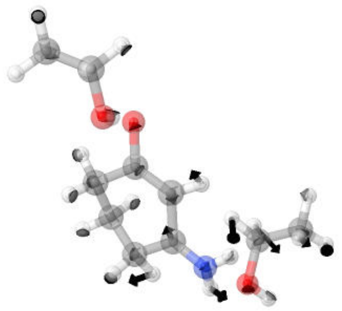
	1663 (24)	H_{16}-N_8-H_{17} scissor 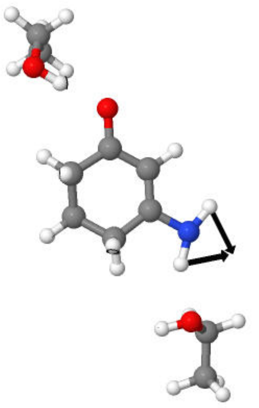	1734 (35)	H_{16}-N_8-H_{17} scissor 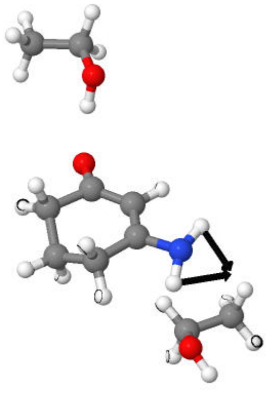

EtOH 2 (0.994)	1559 (315)	C_2=C_3 stretch + C_1=O_7 stretch + C_2-H_9 bend 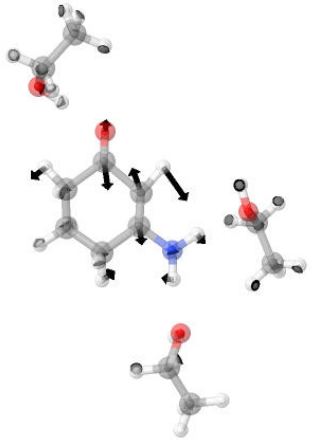		
	1605 (3)	C_2=C_3 stretch + C_1=O_7 stretch + H_{16}-N_8-H_{17} scissor 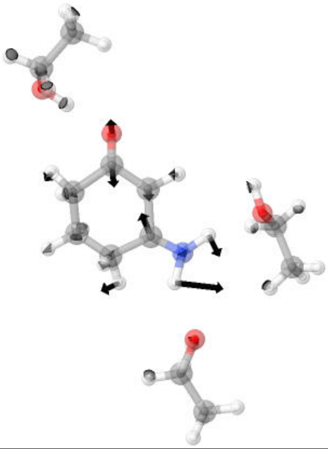		
	1690 (29)	H_{16}-N_8-H_{17} scissor 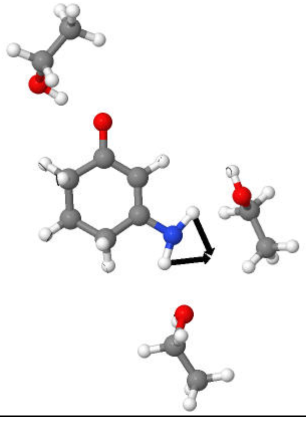		
EtOH 3 (0.999)			1511 (2)	H_{12}-C_5-H_{13} scissor

				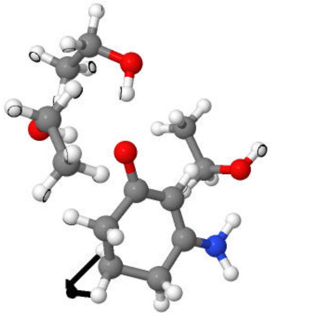
	1559 (283)	C_2=C_3 stretch + C_1=O_7 stretch + C_2-H_9 bend 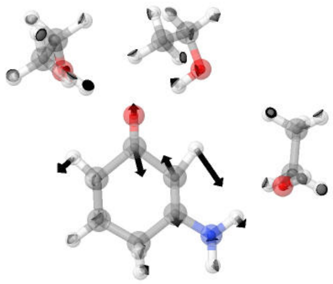	1549 (54)	C_2=C_3 stretch + H_{10}-C_4-H_{11} scissor + N_8-H_{17} bend + C_2-H_9 bend 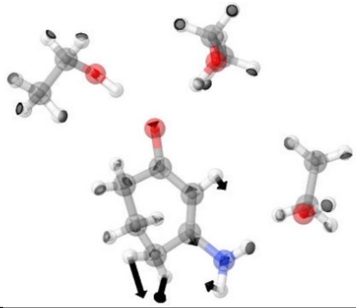
	1607 (4)	C_2=C_3 stretch + C_1=O_7 stretch + H_{16}-N_8-H_{17} scissor 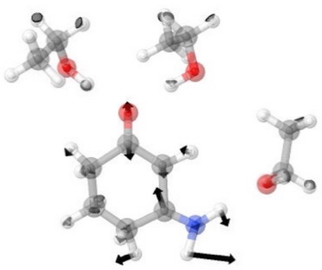	1572 (30)	H_{10}-C_4-H_{11} scissor 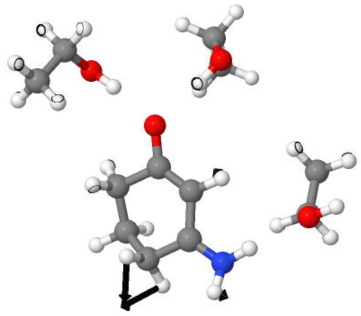
	1685 (26)	H_{16}-N_8-H_{17} scissor 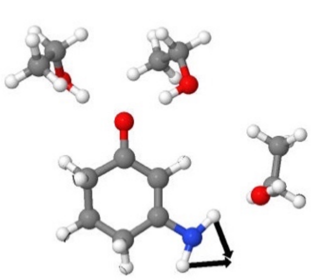	1740 (30)	H_{16}-N_8-H_{17} scissor 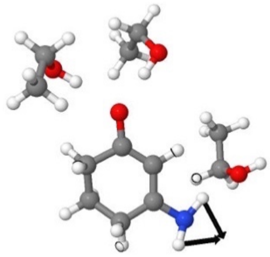
EtOH 4 (1.006)			1520 (7)	H_{10}-C_4-H_{11} scissor

				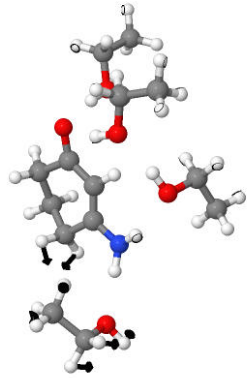
	1559 (317)	C_2=C_3 stretch + C_1=O_7 stretch + C_2-H_9 bend 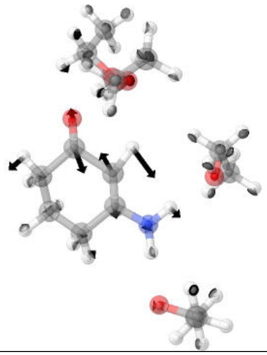	1524 (15)	H_{10}-C_4-H_{11} scissor 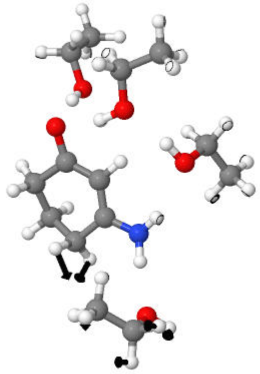
	1612 (8)	C_2=C_3 stretch + C_1=O_7 stretch + H_{16}-N_8-H_{17} scissor 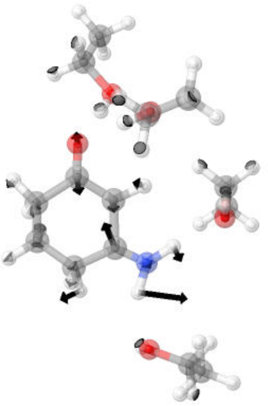	1555 (45)	C_2=C_3 stretch + H_{10}-C_4-H_{11} scissor + N_8-H_{17} bend + C_2-H_9 bend 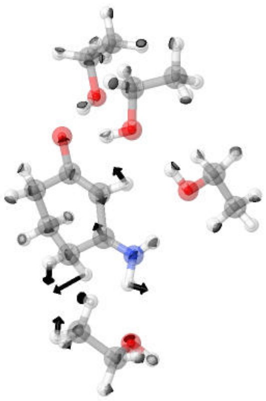
	1707 (30)	H_{16}-N_8-H_{17} scissor 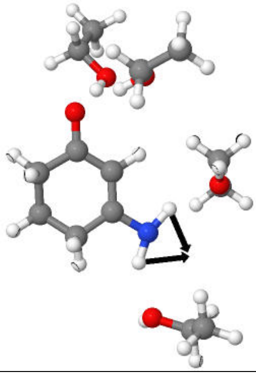	1698 (32)	H_{16}-N_8-H_{17} scissor 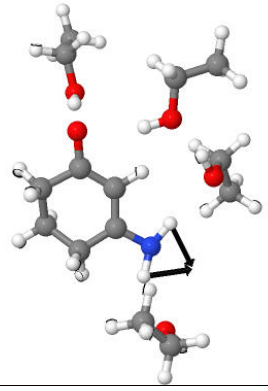

DMSO<br>1<br>(0.982)	1577 (196)	C_2=C_3 stretch + C_4-H_{10} bend + N_8-H_{17} bend + C_2-H_9 bend<br>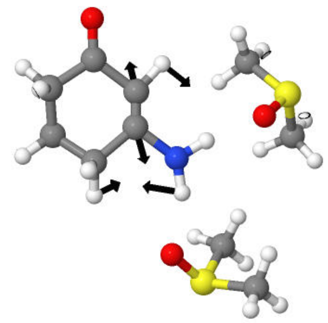	1532 (104)	C_2=C_3 stretch + C_4-H_{10} bend + N_8-H_{17} bend + C_2-H_9 bend<br>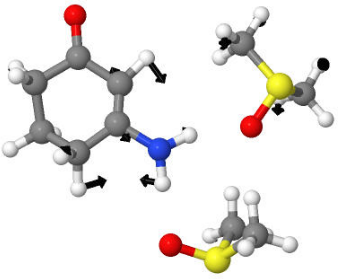
	1648 (109)	C_1=O_7 stretch + H_{16}-N_8-H_{17} scissor<br>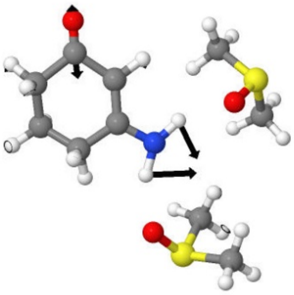		
	1672 (9)	H_{16}-N_8-H_{17} scissor<br>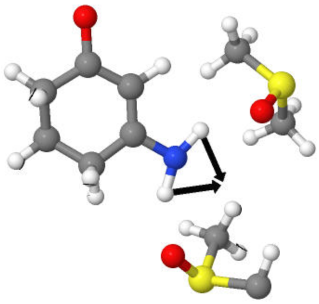	1655 (42)	H_{16}-N_8-H_{17} scissor<br>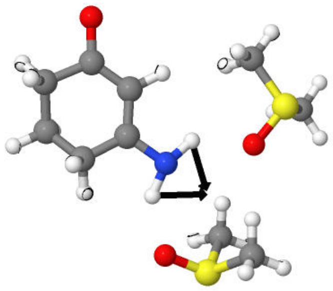
DMSO<br>2<br>(0.983)	1577 (180)	C_2=C_3 stretch + C_4-H_{10} bend + N_8-H_{17} bend + C_2-H_9 bend<br>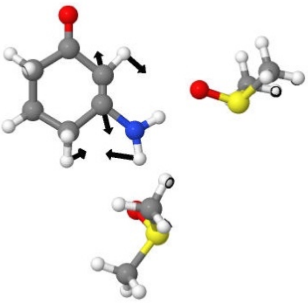	1527 (97)	C_2=C_3 stretch + C_4-H_{10} bend + N_8-H_{17} bend + C_2-H_9 bend<br>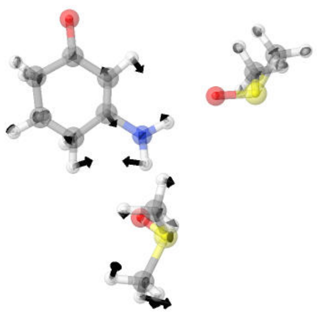
	1647 (116)	C_1=O_7 stretch + H_{16}-N_8-H_{17} scissor		

		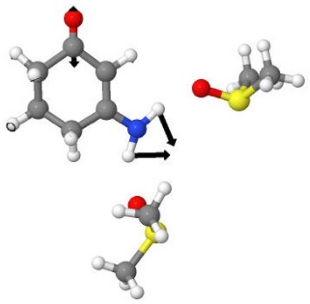		
	1673 (7)	H_{16}-N_8-H_{17} scissor 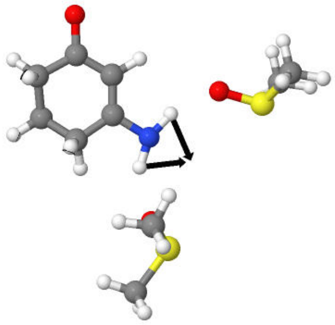	1630 (44)	H_{16}-N_8-H_{17} scissor 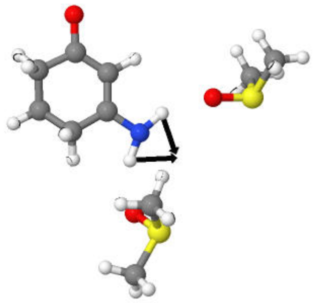
DMSO 3 (0.983)	1577 (187)	C_2=C_3 stretch + C_4-H_{10} bend + N_8-H_{17} bend + C_2-H_9 bend 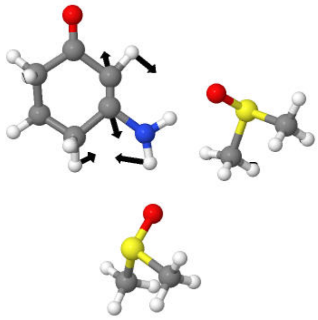	1519 (101)	C_2=C_3 stretch + C_4-H_{10} bend + N_8-H_{17} bend + C_2-H_9 bend 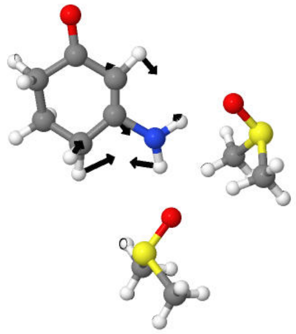
	1650 (111)	C_1=O_7 stretch + H_{16}-N_8-H_{17} scissor 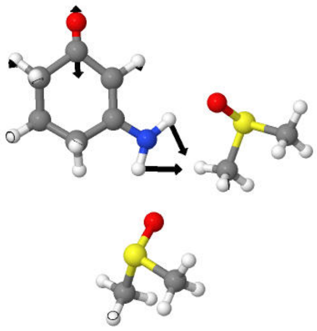		

	1681 (11)	H_{16}-N_8-H_{17} scissor 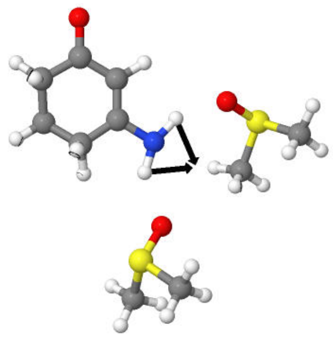	1654 (50)	H_{16}-N_8-H_{17} scissor 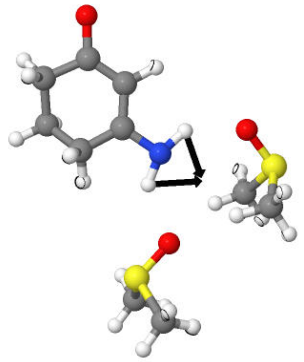
DMSO 4 (0.982)	1577 (176)	C_2=C_3 stretch + C_4-H_{10} bend + N_8-H_{17} bend + C_2-H_9 bend 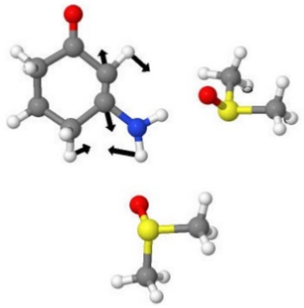	1518 (61)	C_2=C_3 stretch + C_4-H_{10} bend + N_8-H_{17} bend + C_2-H_9 bend 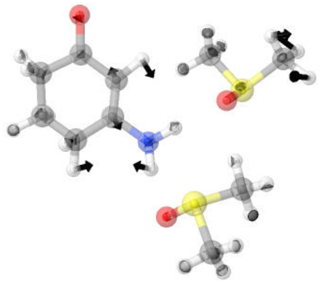
	1648 (108)	C_1=O_7 stretch + H_{16}-N_8-H_{17} scissor 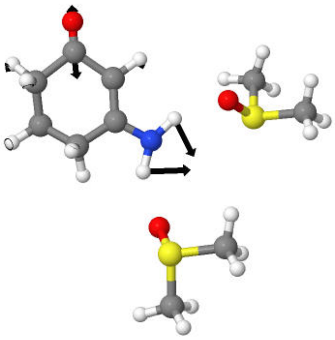		
	1677 (10)	H_{16}-N_8-H_{17} scissor 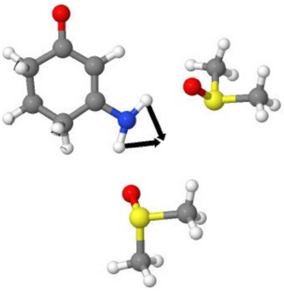	1646 (38)	H_{16}-N_8-H_{17} scissor 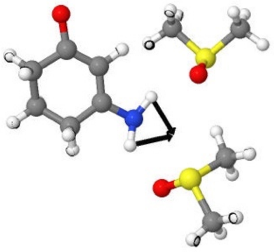

6 TEA spectra lineouts

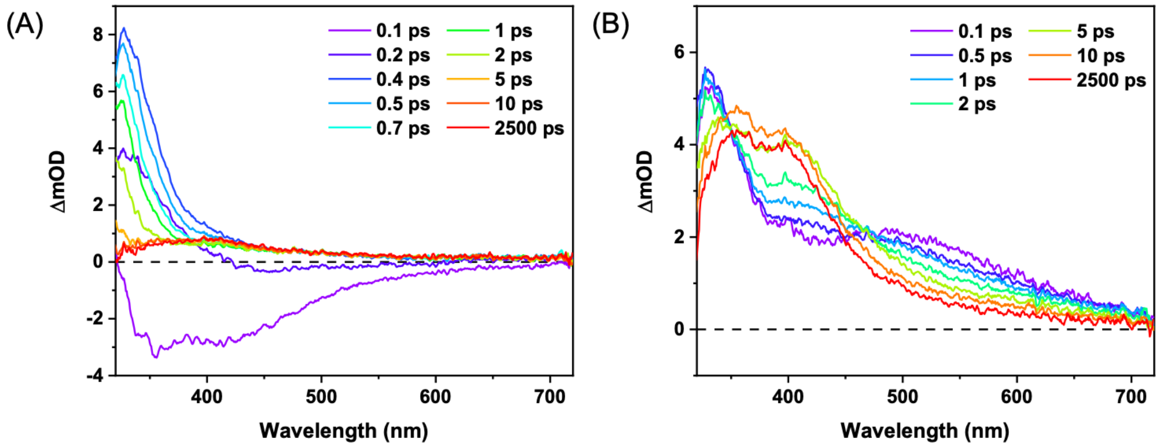

Supplementary Figure 6. TEA spectra at selected time delays for **ACyO** in **(A)** EtOH, photoexcited at 285 nm and **(B)** DMSO, photoexcited at 280 nm.

7 Vertical excitations

Vertical excitations were carried out to check the results are in accordance with the literature which states that **ACyO** is excited to its S_2 state via a $\pi^* \leftarrow \pi$ transition (Sui et al., 2012; Losantos et al., 2017). **Supplementary Table 4** presents the vertical excitation results for the first two singlet states carried out at the PBE0/cc-pVTZ level of theory for all the implicit- and explicit-solvent environments investigated in the present work. **Supplementary Figure 7** is a visual representation of the molecular orbitals associated with the transitions and corroborates the assignment of a $\pi^* \leftarrow \pi$ transition being the most optically bright. Further analysis of the results is outside the scope of the present work.

Supplementary Table 4. PBE0/cc-pVTZ computed vertical excitations and oscillator strengths for all implicit- and explicit-solvent environments investigated in this study up to their second singlet excited state.

Solvent model	Singlet state	Orbital character	Transition energies, nm (oscillator strength *f*)
**Implicit**			
EtOH	S_1	$\pi^*_L \leftarrow n_{H-1}$	277 (0.0010)
	S_2	$\pi^*_L \leftarrow \pi_H$	233 (0.4430)
DMSO	S_1	$\pi^*_L \leftarrow n_{H-1}$	289 (0.0005)
	S_2	$\pi^*_L \leftarrow \pi_H$	231 (0.4422)
**Explicit**			
EtOH 1	S_1	$\pi^*_L \leftarrow n_{H-1}$	266 (0.0059)
	S_2	$\pi^*_L \leftarrow \pi_H$	238 (0.6225)
EtOH 2	S_1	$\pi^*_L \leftarrow n_{H-1}$	265 (0.0052)
	S_2	$\pi^*_L \leftarrow \pi_H$	240 (0.6338)
EtOH 3	S_1	$\pi^*_L \leftarrow n_{H-1}$	260 (0.0086)
	S_2	$\pi^*_L \leftarrow \pi_H$	240 (0.5236)
EtOH 4	S_1	$\pi^*_L \leftarrow n_{H-1}$	263 (0.0083)
	S_2	$\pi^*_L \leftarrow \pi_H$	245 (0.5405)
DMSO 1	S_1	$\pi^*_L \leftarrow n_{H-1}$	291 (0.0008)
	S_2	$\pi^*_L \leftarrow \pi_H$	242 (0.5166)
DMSO 2	S_1	$\pi^*_L \leftarrow n_{H-1}$	291 (0.0011)
	S_2	$\pi^*_L \leftarrow \pi_H$	241 (0.5442)
DMSO 3	S_1	$\pi^*_L \leftarrow n_{H-1}$	291 (0.0010)
	S_2	$\pi^*_L \leftarrow \pi_H$	241 (0.5880)
DMSO 4	S_1	$\pi^*_L \leftarrow n_{H-1}$	291 (0.0012)
	S_2	$\pi^*_L \leftarrow \pi_H$	241 (0.5689)

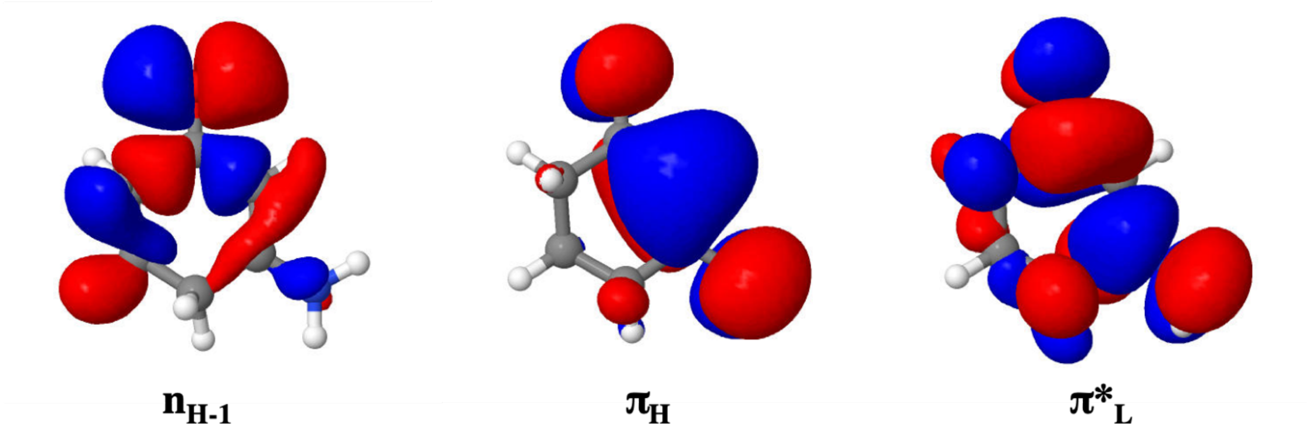

Supplementary Figure 7. Molecular orbitals of **ACyO** involved in the first two singlet state transitions.

8 Long-term irradiation control study

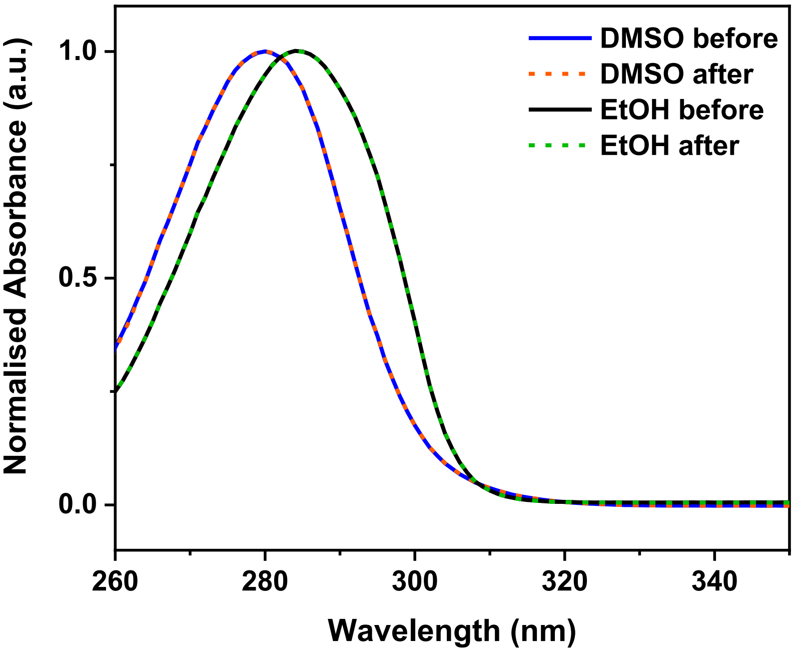

Supplementary Figure 8. UV/visible spectra of ~40 µM **ACyO** in EtOH and DMSO taken before and after 7200 s without irradiation.

9 Implicit-solvent geometry optimisation structures and their energy

Supplementary Table 5. Starting implicit-solvent **ACyO** structures and their corresponding S_0 and S_1 relaxed geometry computed at the PBE0/cc-pVTZ level of theory. Also reported are the relative energies of the S_1 relaxed geometries with respect to their S_0 relaxed geometries.

**Before geometry optimisation**	**S_0 relaxed geometry and relative energy**	**S_1 relaxed geometry and relative energy**
EtOH 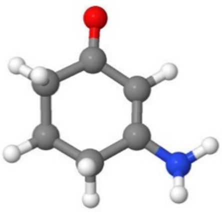	0 eV 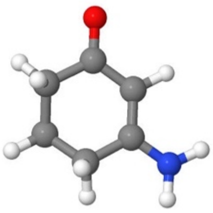	4.3329636 eV 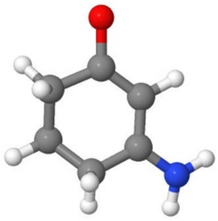
DMSO 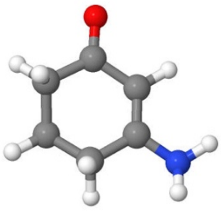	0 eV 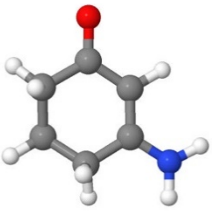	4.0632796 eV 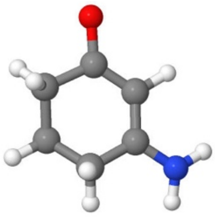

Supplementary Material References

Losantos, R., Funes-Ardoiz, I., Aguilera, J., Herrera-Ceballos, E., Garcia-Iriepa, C., Campos, P.J., et al. (2017). Rational Design and Synthesis of Efficient Sunscreens To Boost the Solar Protection Factor. *Angew. Chem. Int. Ed.* 56**,** 2632-2635. doi: 10.1002/anie.201611627.

Sui, X.-X., Li, L., Zhao, Y., Wang, H.-G., Pei, K.-M., and Zheng, X. (2012). Resonance Raman and density functional study of the excited state structural dynamics of 3-amino-2-cyclohexen-1-one in water and acetonitrile solvents. *Spectrochim. Acta A* 85**,** 165-172. doi: 10.1016/j.saa.2011.09.055.
